# Engineering Functional PVA: A Comprehensive Review of Chemical Modifications and Prospective Developments

**DOI:** 10.1021/acspolymersau.5c00133

**Published:** 2025-11-17

**Authors:** Nhu Q. Vu, Thai M. Le, An N. B. Ngo, Minh H. T. Nguyen, Ying-Chih Liao, Thuy T. Tran

**Affiliations:** † 118018 Hanoi University of Science and Technology, 1 Dai Co Viet Street, 100000 Hanoi, Vietnam; ‡ Department of Chemical Engineering, 33561 National Taiwan University, 10617 Taipei, Taiwan

**Keywords:** poly(vinyl alcohol) (PVA), chemical modification, cross-linking, grafting, functionalization, esterification, acetalization, urethanation, sulfonation

## Abstract

In response to the growing demand for biocompatible and mechanically resilient materials, poly­(vinyl alcohol) (PVA)-based systems have gained considerable attention for their versatility in biomedical, industrial, and environmental uses. While PVA exhibits excellent film-forming and hydrogel-forming capabilities, as well as great biocompatibility, emerging applications require enhancements in thermal and mechanical performance, along with eco-friendly processing. This work identifies key strategies to augment PVA with desirable qualities, namely, cross-linking, grafting, and functionalization. For each approach, the underlying reaction mechanisms, critical processing parameters, and post-treatment effects are discussed in detail. The functional outcomes of these modifications are evaluated in the context of real-world applications, demonstrating PVA’s adaptability and performance potential. Finally, obstacles impeding the development of PVA materials are addressed, along with noteworthy breakthroughs found in the recent literature. Future directions in research are also proposed to both leverage these newfound advancements and overcome the current hardships to further remarkable developments in practical utilizations.

## Graphical abstract

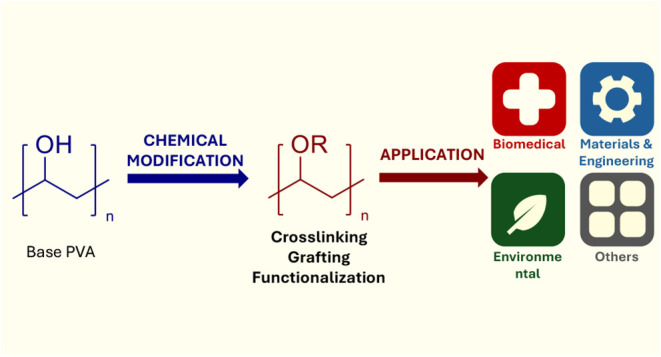

## Introduction

Poly­(vinyl alcohol) (PVA) has garnered significant attention in various advanced industries owing to its unique combination of properties, including excellent water solubility, tunable mechanical strength, film-forming ability, and remarkable biocompatibility.^1–3^ These characteristics render PVA highly suitable for a wide range of applications, spanning biomedical devices, environmental remediation, packaging materials, and energy-related technologies.

Amid growing environmental concerns and the global push toward sustainable development, the demand for biodegradable and eco-friendly polymers is rapidly increasing. In this context, PVA stands out as a promising candidate due to its potential for biodegradability under suitable environmental conditions, creating new opportunities for sustainable and green material solutions.^4–6^

PVA is not synthesized directly from vinyl alcohol monomers, which are unstable in their free form. Instead, it is obtained through the hydrolysis of poly­(vinyl acetate) (PVAc), a process that can be catalyzed by either acids or bases.^7^ The degree of hydrolysis significantly influences the final properties of PVA, leading to fully or partially hydrolyzed forms with distinct water solubility and mechanical behaviors.^7,8^ Figure 1 presents the reaction scheme illustrating the synthesis of PVA from vinyl acetate.

**Figure 1:**
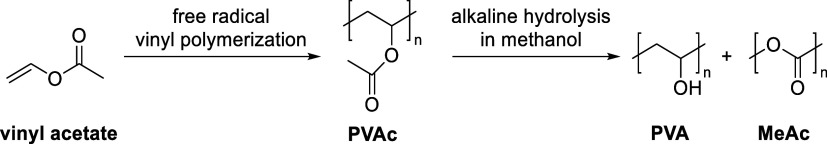
Reaction scheme for the synthesis of PVA from vinyl acetate.

Structurally, PVA is a linear, semicrystalline polymer containing abundant hydroxyl (−OH) groups along its backbone. These functional groups enable strong intra- and intermolecular hydrogen bonding, which plays a pivotal role in defining the polymer’s solubility, mechanical integrity, and biocompatibility.^9,10^ Moreover, the presence of hydroxyl groups offers numerous opportunities for chemical modifications to enhance the thermal stability, water resistance, and strength.

The physical properties of PVA are strongly influenced by its degree of hydrolysis and molecular weight. A higher degree of hydrolysis increases the crystallinity, tensile strength, and resistance to water and solvents. However, as the hydrolysis degree rises, the polymer also becomes stiffer, and both the melting temperature and glass transition temperature (*T*_g_) increase, while the free volume decreases.^11–13^ However, higher molecular weight is typically associated with greater tensile strength and elastic modulus.^14–16^ Balancing these opposing factors is essential for tailoring PVA for specific applications. Despite these advantages, pristine PVA tends to be brittle and susceptible to fracture under mechanical stress.

PVA’s thermal stability is limited and varies significantly with phase. It melts at around 230 °C (crystallinity ≈ 65%) and, above this temperature, undergoes rapid chain scission-predominantly losing water and small carbonyl compounds, resulting in a mass loss of approximately 80–90% by 300 °C.^17^ Below its melting point, degradation is mainly via a four-membered ring dehydration reaction (modest mass loss at ≈195 °C) with much slower kinetics.

Thanks to its favorable physicochemical properties, PVA has found extensive applications in biodegradable packaging, textiles, adhesives, coatings, and water treatment. In the biomedical domain, it is utilized in contact lenses,^18,19^ wound dressings,^20^ drug-delivery systems,^21,22^ and tissue-engineering scaffolds.^23^ Furthermore, PVA’s hydrogel-forming capability has made it a valuable material for biosensors,^24–26^ fuel cells,^27^ and flexible electronics.^28,29^ Efforts are also underway to develop PVA-based alternatives to traditional plastics, reflecting their potential in sustainable materials development.

Nevertheless, PVA faces several limitations that hinder its broader applicability, including limited barrier and thermal properties, relatively high cost, inert bioactivity, low elasticity, and restricted functionality in biomedical applications.^10,30^ To address these challenges and broaden the utility of PVA, chemical modification is a powerful strategy to tailor its properties without compromising its inherent advantages. This review aims to provide a comprehensive overview of the chemical modification methods applied to PVA, with a particular emphasis on cross-linking, grafting, and functionalization strategies. Functionalization techniques discussed include esterification, acetalization, urethanation, sulfonation, and other advanced chemical modifications. This review also highlights the current challenges, recent breakthroughs, and future directions aimed at further enhancing the mechanical, electrical, and biological properties of this polymer.

## Chemical Modification Strategies of PVA

### Cross-linking

Cross-linking is a fundamental process employed to engineer three-dimensional networks within PVA by establishing interchain interactions or bonds. Depending on the nature of the bonds formed, cross-linking methods can be classified into physical, chemical, and hybrid cross-linking. In physical cross-linking, noncovalent interactions such as hydrogen bonding and the formation of crystalline domains are induced through methods like thermal annealing or repeated freeze–thaw cycles, resulting in a reversible polymer network. Chemical cross-linking, in contrast, involves the formation of permanent covalent bonds between polymer chains, typically facilitated by the addition of cross-linking agents or exposure to high-energy irradiation, thereby yielding structures with enhanced stability and durability. Hybrid cross-linking represents a synergistic approach, integrating both physical and chemical strategies to fabricate materials that harness the benefits of each method. This combined approach enables precise tailoring of key properties, including mechanical strength, thermal resistance, and biodegradability.

Consequently, cross-linking serves as a highly effective strategy for augmenting the performance and expanding the functional versatility of PVA-based materials across a wide spectrum of applications.

#### Chemical Cross-linking

Chemical cross-linking of PVA involves the formation of permanent covalent bonds between polymer chains through reactions with cross-linking agents such as aldehydes, organic acids, inorganic acids, expoxides, and diisocyanates.

This process enhances the structural integrity of PVA by replacing weak, reversible physical interactions with stable chemical linkages, leading to a more robust polymer network.^31^ The reaction primarily occurs between the hydroxyl (−OH) groups of PVA and the reactive functional groups of the cross-linking agents.

For instance, glutaraldehyde (GA) and citric acid (CA) both act as cross-linking agents that chemically bond with PVA chains GA forms acetal linkages through reactions with hydroxyl groups, whereas citric acid forms ester bonds via condensation reactions. These irreversible covalent bonds significantly improve the material’s mechanical strength, thermal stability, and water resistance. Moreover, precise control over the cross-linking agent concentration allows for the fine-tuning of cross-linking density, enabling the fabrication of materials with tailored properties for diverse applications.^32^

#### Effect of Processing Conditions on Crosslinked PVA

The properties of chemically crosslinked PVA are highly influenced by processing conditions, including temperature, reaction time, and pH. Higher cross-linking temperatures and prolonged reaction times typically increase cross-linking density, leading to enhanced mechanical strength and thermal stability.^33^ However, excessive cross-linking can increase the material’s brittleness and reduce flexibility, potentially limiting its applicability in certain fields. Conversely, lower temperatures and shorter reaction times result in materials with lower cross-linking density, offering greater flexibility but reduced mechanical robustness.^33^ Additionally, the pH of the reaction medium plays a crucial role in cross-linking efficiency, particularly for agents like citric acid, where esterification reactions are highly pH dependent.^31^ Therefore, optimizing these parameters is essential to achieving the desired balance between mechanical performance and flexibility.^31,34^

To illustrate how processing conditions influence material properties, Table 1 below compares GA cross-linking under different parameters. This comparison highlights the critical role of reaction parameters in determining the cross-linking density, mechanical strength, and thermal stability.

**Table 1:** Influence of Environmental Conditions on the Properties of PVA–GA Crosslinked Materials

Parameters	PVA-GA (1%)	PVA-GA (2%)	PVA-GA (4%)	PVA-GA (6%)	PVA-GA (1.57%)	PVA-GA (20%)
GA Concentration (w/w)	1%	2%	4%	6%	1.57%	20%
Cross-linking Temperature (°C)	Not mentioned	Not mentioned	Not mentioned	Not mentioned	80 °C	50 °C
Duration of Cross-linking	Not mentioned	Not mentioned	Not mentioned	Not mentioned	2 h	6 to 48 h
Degree of Cross-linking (XRD)	2.8%	5.6%	32%	34%	Not mentioned	Not mentioned
Tensile strength (MPa)	∼9–10	∼14–15	30	∼18	Not mentioned	4.44 (neat PVA) 12.70 (crosslinked)
Proton conductivity (S/cm)	7.53 × 10^–3^	8.4 × 10^–4^	1.2 × 10^–4^	4.5 × 10^–5^	Not mentioned	Not mentioned
Elongation (%)	∼200%	∼150%	45.2%	40%	Not mentioned	56.19% (neat PVA), 24.14% (crosslinked)
Swelling ratio (%)	275%	183%	70%	56%	<80%	None
Water uptake (%)	515%	292%	142%	132%	Reduce by 30–50% in water	Not mentioned
Applications	Water filtration and wastewater treatment	Microbial Fuel Cells (MFCs), water filtration systems	MFCs for enhanced electrical performance	MFCs with high stability, separation membranes	Vitreous replacement	Antimicrobial agent, wound dressing, water/air purification
ref	^35^	^35^	^35^	^35^	^36^	^37^

#### Cross-linking Agents for PVA: Classification and Properties

To provide a comprehensive overview of the strategies used to enhance the structural stability and functionality of PVA-based materials, Table 2 summarizes various classes of cross-linking agents commonly applied to PVA systems. This classification helps to highlight not only the technical performance but also the biocompatibility and ecological aspects critical for selecting cross-linkers, especially in biomedical and environmentally sensitive applications.

**Table 2:**
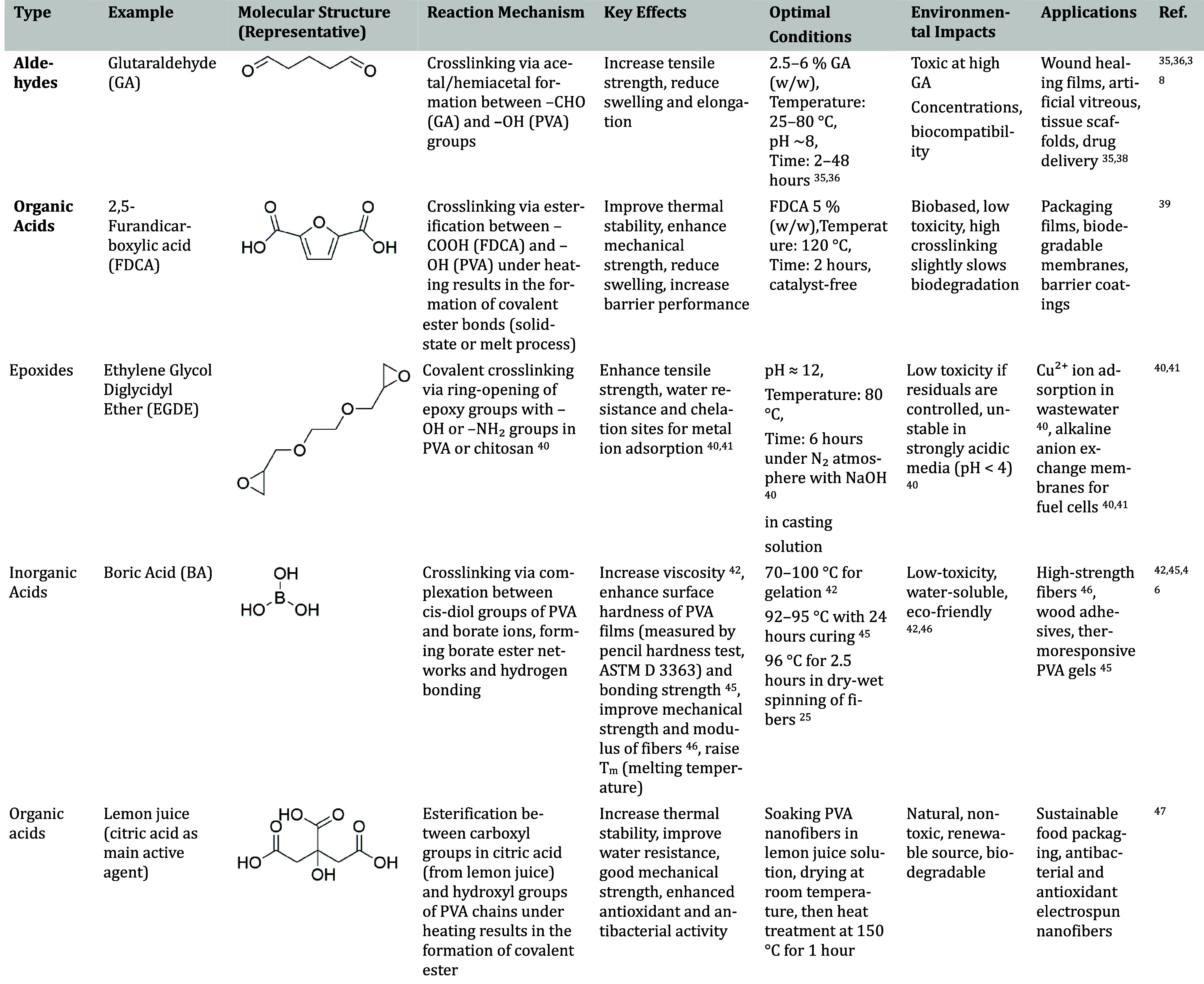
Classification of Cross-linking Agents for PVA^42–44^

The comparison of different cross-linking agents for PVA shows that chemical and natural cross-linking are not entirely separate approaches but have a complementary and interdependent relationship. In many cases, natural cross-linkers serve both as active agents and as catalysts that promote chemical cross-linking reactions under mild conditions.

Traditional chemical agents such as GA are effective in reinforcing the network structure by improving tensile strength, swelling resistance, and thermal stability; however, their high cytotoxicity and the need for complex purification limit their biomedical applicability. In contrast, natural acidic cross-linkers such as pure lemon juice not only provide biocompatibility and environmental safety but also serve as reactive components in the chemical cross-linking process. The citric acid contained in lemon juice can catalyze esterification reactions with PVA hydroxyl groups, forming stable covalent bonds without the introduction of toxic residues.

This dual role allows natural cross-linkers to enhance the effectiveness of chemical cross-linking while maintaining good mechanical properties, high water resistance, and excellent biological performance. Therefore, rather than being viewed as independent strategies, natural and chemical cross-linking should be considered as synergistic processes that can be combined to optimize the structure, functionality, and safety of PVA-based materials for biomedical and environmental applications.

##### Aldehydes

PVA cross-linking with GA is typically performed under acid-catalyzed conditions to produce acetal and ether-type bridges between PVA hydroxyls, giving an insoluble, dimensionally stable network.^35^ In practice, PVA is cast from aqueous solution (e.g., 3% w/v), then mixed with controlled amounts of GA (the authors report additions of 0.1–0.6 mL of 25% GA solution, producing membranes denoted PVA_1_–PVA_4_) together with a small amount of H_2_SO_4_ as the catalyst, acetic acid as the pH controller and methanol as a quencher.^35^ After casting and drying, the gel fraction (degree of cross-linking) rises steeply with GA content (≈2.8, 5.6, 32, and 34% for the four formulations).^35^ Increasing GA produces clear, systematic property changes: water uptake falls (reported values 515 → 292 → 142 → 132%), swelling ratios drop (275 → 183 → 70 → 56%), and crystallinity is reduced; FTIR shows decreased – OH intensity with the appearance/shift of C–O–C and acetal bands, consistent with acetal/ether formation.^35^ Thermal stability and tensile strength increase up to an optimum (tensile strength reported ≈30 MPa for the midcross-linked sample), while proton conductivity and ion-exchange capacity decrease because the tighter network restricts ionic mobility.^35^ These acid-catalyzed GA-crosslinked PVA membranes therefore trade higher mechanical/thermal stability and lower oxygen permeability for reduced water uptake and ionic transport-features that make them useful where dimensional stability and reduced swelling are required (e.g., membrane separators), but less suitable when high ionic conductivity or extreme acid stability is needed.^35^

The GA cross-linking protocol for hybrid biomaterial films based on HAP/PVA/gelatin is generally mild. Typically, about 2.5% GA is added at 40–50 °C with brief stirring, followed by curing and drying of the films.^38^ This method is often combined with plasticizers such as glycerin to enhance flexibility and smoothen the film surface for biomedical applications.^38^ The resulting crosslinked films show good structural integrity, reduced solubility, and improved tensile strength and thermal stability compared with un-crosslinked PVA-based films, confirming the effective formation of acetal linkages between GA and hydroxyl groups.^38^ Glutaraldehyde concentrations up to 8% are generally acceptable for such uses, although cytotoxicity depends on the dosage and post-treatment conditions.^38^

##### Organic Acids

Organic acids such as citric acid, maleic acid, succinic acid, and oxalic acid have long been recognized as effective green cross-linking agents for PVA due to their multifunctional carboxyl groups, which can form ester bonds with the hydroxyl groups of PVA upon heating. This esterification reaction typically proceeds under acidic or dehydrating conditions, producing a three-dimensional covalent network that improves the film’s water resistance, mechanical integrity, and thermal stability. The resulting crosslinked structures retain partial hydrophilicity owing to unreacted carboxyl groups, which can further promote ion-exchange or metal ion adsorption in environmental or biomedical applications.

To better understand how different carboxylic acids influence the physical properties of crosslinked PVA materials, four representative dicarboxylic acids, sucinic, malic, maleic, and aspartic, were compared under the same preparation conditions. As summarized in Table 3, succinic acid (PSAH) exhibited the highest tensile strength and elongation, while aspartic acid (PAAH) showed the highest swelling ratio but lower mechanical stability. The use of malic and maleic acids produced intermediate properties with a balance between flexibility and stiffness.

**Table 3:**
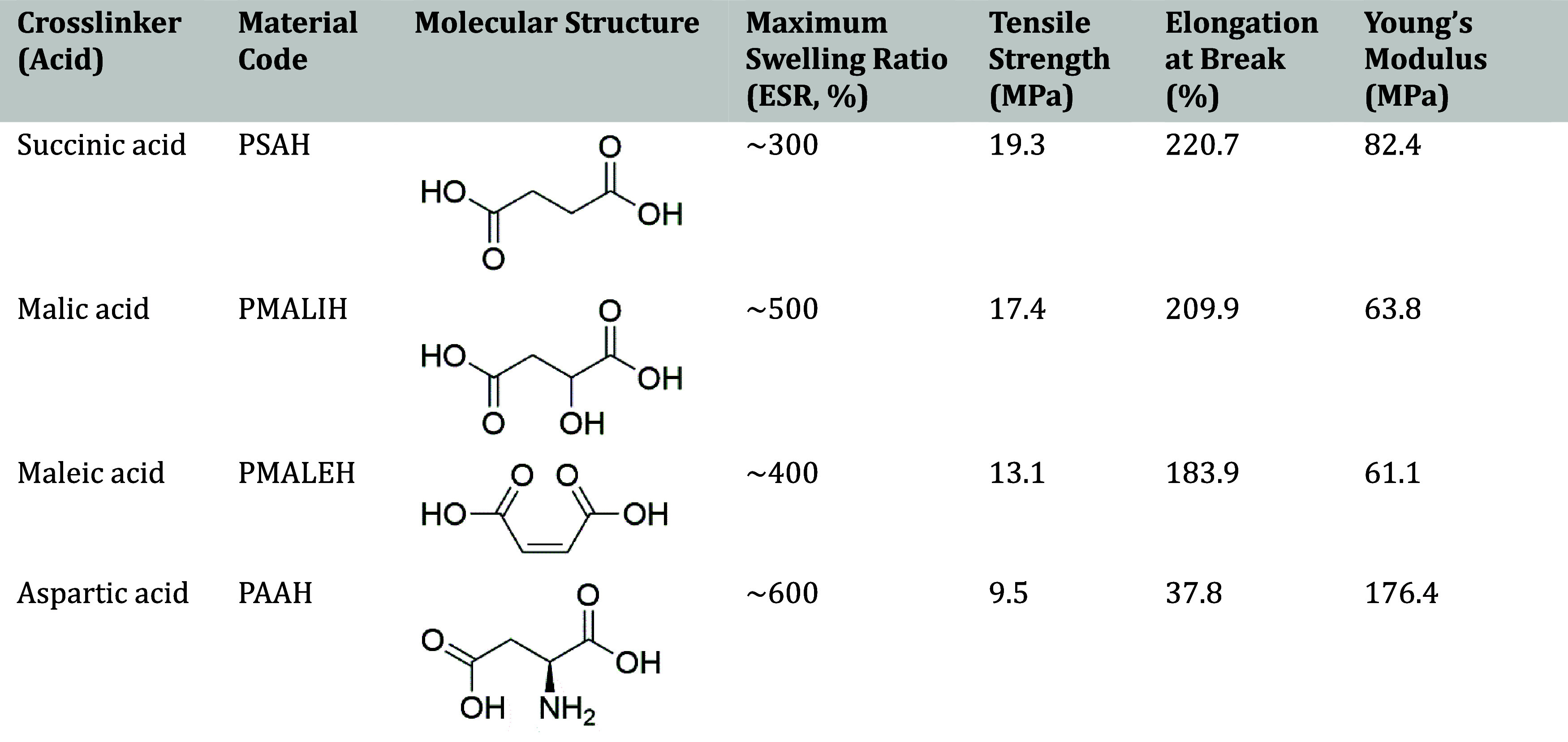
Comparison of Physicochemical and Mechanical Properties of Crosslinked PVA Materials with Different Carboxylic Acids^49^

2,5-Furandicarboxylic acid (FDCA), a biobased cross-linking agent, has attracted considerable attention in recent years due to its ability to enhance the properties of sustainable materials. Nam et al. (2022) investigated the use of FDCA to cross-link PVA through a catalyst-free solid-state esterification reaction at 120 °C. The results showed that FDCA significantly reduced the water absorption of PVA (from 98.46% to 35.57% at 10% FDCA concentration), while increasing the maximum decomposition temperature from 267 to 371 °C and doubling the tensile strength (48.2 MPa compared to 25.5 MPa for neat PVA) at a 5% FDCA concentration.^39^ By 2024, Ni et al. had investigated the effect of cellulose nanofibers on PVA crosslinked with FDCA and reported that with a nano cellulose fiber (CNF) content of 2%, the tensile strength doubled (37.08 MPa) compared to unmodified PVA (14.57 MPa), while the decomposition temperature increased to 371 °C.^48^

The main applications of carboxylic acid-crosslinked PVA are summarized in Table 4, providing an overview of their practical uses across different areas.

**Table 4:**
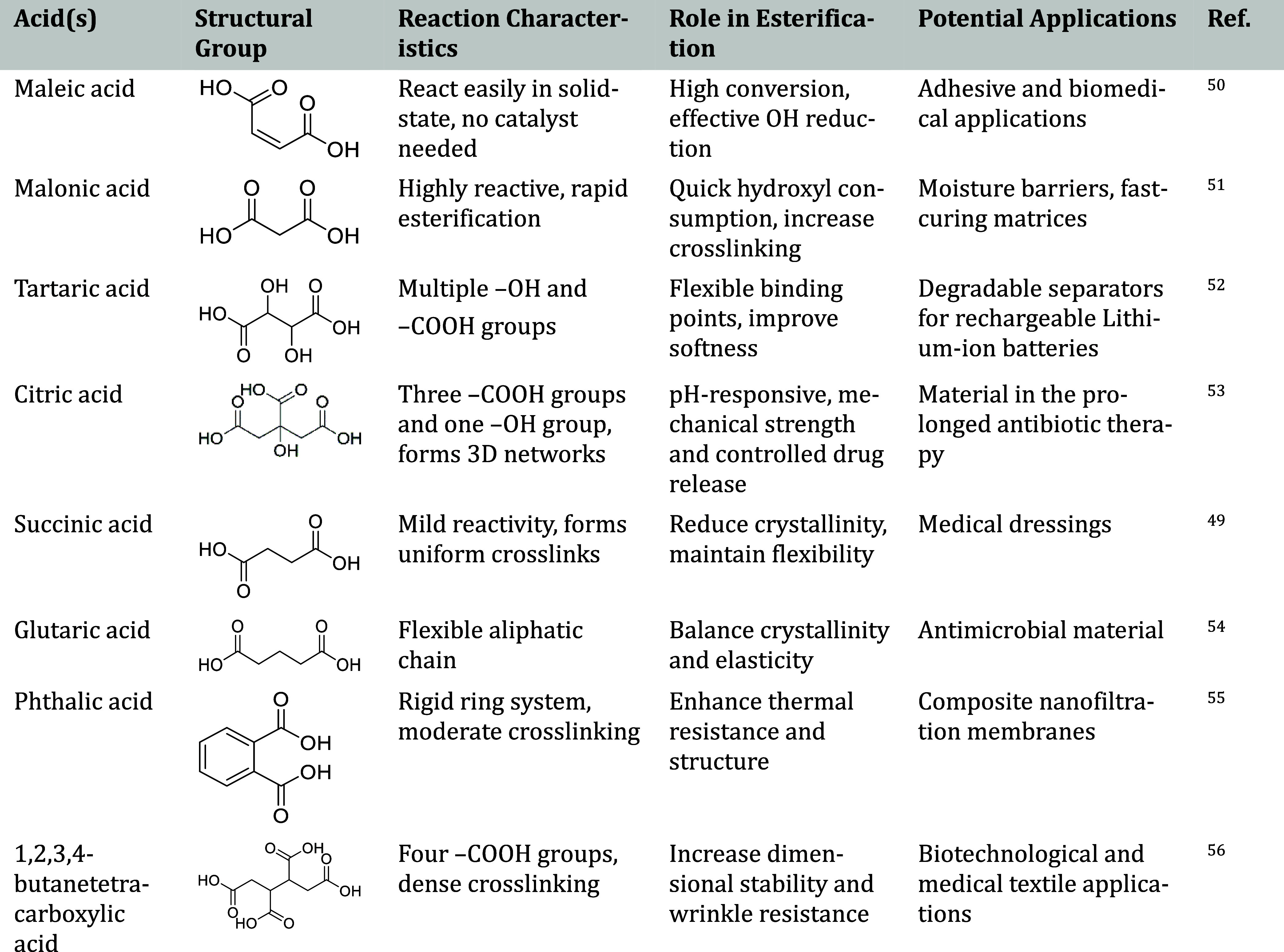
Main Applications of Carboxylic Acid-Crosslinked PVA

Building upon these advances in biobased cross-linking chemistry, recent studies have further explored the use of natural acidic sources such as lemon juice, where citric acid serves as the active cross-linking component for PVA, enabling a fully green and sustainable esterification process.^47^ Electrospun PVA nanofibrous mats were prepared using lemon juice as the cross-linking medium and subsequently stabilized at 60 °C for 7 days, promoting low-temperature esterification between the hydroxyl groups of PVA and the carboxyl groups of citric acid. This reaction generated covalent ester bonds that formed a three-dimensional network, improving the nanofibers’ water resistance while preserving their flexibility and morphology.

FTIR spectra confirmed the formation of ester linkages by the appearance of a new band at 1720 cm^–1^ (C=O stretching) and a reduction of O–H intensity. TGA and DSC analyses indicated higher thermal stability and restricted chain mobility, while XRD patterns showed decreased crystallinity due to network formation. The optimized sample (L/60C) exhibited enhanced mechanical strength and higher Young’s modulus, as well as antioxidant and antibacterial activities, attributed to the bioactive compounds in lemon juice. Overall, lemon-juice-induced cross-linking provides a nontoxic, renewable, and efficient green approach to improve the structural and functional performance of PVA-based materials.^47^

##### Epoxides

Epoxides such as ethylene glycol diglycidyl ether (EGDE) have been extensively employed as efficient cross-linking agents for PVA due to their ability to form stable ether linkages with hydroxyl groups on the PVA backbone. Upon ring-opening of the epoxy groups under mild basic or thermal conditions, covalent C–O–C bridges are created between polymer chains, resulting in an insoluble and mechanically robust network. A PVA-containing formulation (here as Ch/PVA beads or blended films) can be rendered insoluble and dimensionally stable by covalent cross-linking with EGDE. In practice, the Ch/PVA mixture is dripped into a basic bath, and the wet beads are thermally reacted with EGDE under nitrogen at about 353 K to form a network in which the diglycidyl ether reacts with hydroxyl (and amino) groups to produce ether linkages that reduce chain mobility and swelling while retaining some semicrystalline character. Under these conditions, the EGDE-crosslinked material shows markedly improved resistance to neutral and mildly acidic media (stable above pH ≈ 4) and excellent Cu­(II) uptake (Langmuir *q*_max_ reported), but it is not compatible with very strong acids; extensive mass loss and fragmentation occur at pH = 1.^40^

More generally, PVA’s many hydroxyl sites make it particularly amenable to such diglycidyl ether cross-linking, which is widely used to restrain swelling and improve mechanical/chemical stability in PVA-based adsorbents and membranes; reviews of PVA cross-linking note EGDE among common covalent cross-linkers used to tune water uptake, tensile strength, and chemical resilience.^41^

##### Inorganic Acids

Among various inorganic acids used for PVA modification, boric acid (BA) has been widely recognized as an effective cross-linking agent because of its ability to form reversible covalent bonds with hydroxyl groups, improving water resistance and mechanical stability of PVA-based materials. Cross-linking between PVA and boric acid forms didiol complexes and hydrogen bonds that significantly enhance mechanical and thermal performance. In the work of Gadhave et al. (2019),^45^ different hydrolyzed grades of PVA (partially, fully, and modified) were reacted with boric acid (0–1.5 wt %) at 92–95 °C for 2.5 h. The addition of boric acid increased viscosity from 271 poise to 600 poise, glass transition temperature (*T*_g_) from 72 to 87 °C, and tensile strength from 47.2 to 70.4 kg/cm^2^, confirming effective cross-link formation between −OH groups of PVA and borate ions. Similarly, Hong et al. (2021)^46^ prepared dry–wet spun PVA fibers crosslinked with 0.1–0.4 wt % boric acid. Fourier transform infrared spectra showed a shift of the −OH peak to higher wavenumbers, indicating the formation of B–O–C bonds and reduced hydrogen bonding. The PVA/BA–0.3 wt % fiber exhibited the best performance, with a tensile strength of 13.1 ± 0.4 cN/dtex, Young’s modulus of 360.2 ± 10.4 cN/dtex, and crystallinity of 50.4%. These results confirm that boric acid efficiently enhances PVA’s network structure, improving both mechanical strength and thermal stability.^45,46^

##### Diisocyanate

The cross-linking of PVA with diisocyanates primarily occurs through the nucleophilic addition of hydroxyl groups (−OH) in PVA to isocyanate groups (−NCO), forming urethane linkages (−NH–CO–O−). This reaction establishes covalent cross-links within the PVA network, significantly improving its mechanical strength, chemical resistance, and thermal stability. The extent and nature of cross-linking depend on the type of diisocyanate, reaction conditions, and reactant ratios. Consequently, diisocyanate-crosslinked PVA materials are widely applied in packaging, biomedical engineering, and functional coatings.

A representative case is interfacial polymerization, where PVA is dissolved in the aqueous phase while diisocyanates (e.g., TDI) are introduced from an organic phase, promoting efficient urethane bond formation at the phase boundary,^57^ as illustrated in Figure 2.

**Figure 2:**
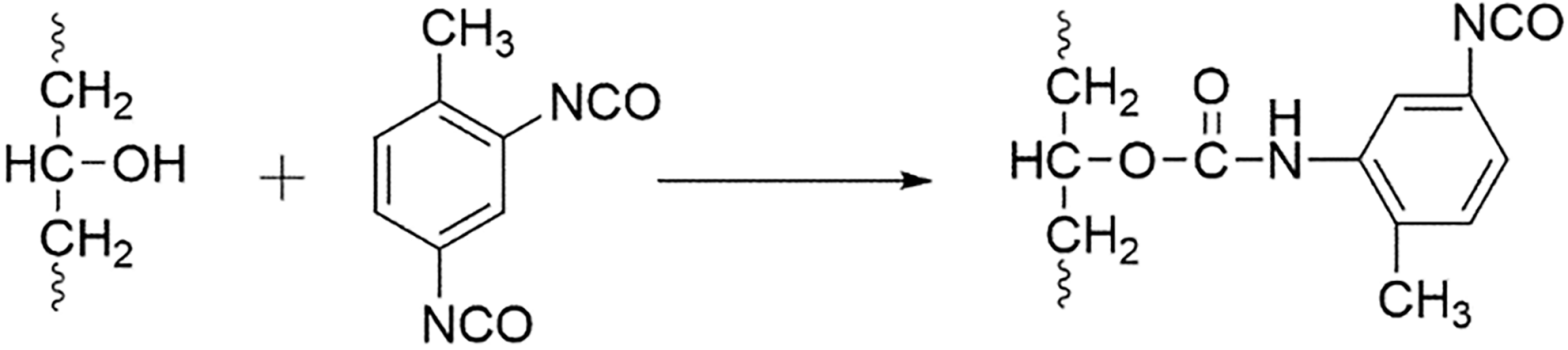
Interfacial polymerization reaction between PVA and TDI.

Common catalysts such as dibutyltin dilaurate (DBTDL),^62^ bismuth-based compounds^58^ promote the cross-linking reaction under mild conditions or with sterically hindered isocyanates. In some cases, the reaction proceeds without catalysts in polar solvents like dimethyl sulfoxide (DMSO) or DMSO/water mixtures, which enhance solubility and reaction kinetics.^59^ Experimental conditions vary widely (60–170 °C) depending on the isocyanate and catalyst used. When multifunctional isocyanates or water as a blowing agent are involved, urea linkages may also form, improving the material’s strength and water resistance.^60^ Table 5 provides a summary of commonly used urethanation agents along with their respective reaction conditions in the modification of PVA.

**Table 5:**
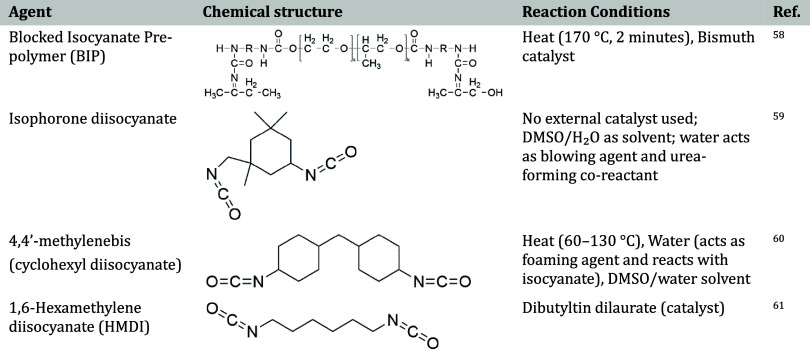
Summary of Isocyanate Agents for PVA Cross-linking and Reaction Conditions

##### Challenges and Prospects of PVA Cross-linking Strategies

A wide range of chemical agents has been employed to cross-link PVA, each offering different reactivity and stability profiles. While these agents can effectively enhance mechanical strength, water resistance, and thermal stability, many of them pose environmental or biocompatibility concerns due to the release of toxic byproducts or harsh reaction conditions. Therefore, there has been a growing interest in replacing these traditional agents with natural and biobased cross-linkers such as citric acid, genipin, or tannic acid, which are safer and more sustainable alternatives. Despite these advantages, natural cross-linkers also present challenges. Optimizing cross-linking conditions, such as concentration, reaction time, and temperature, is crucial to achieving the desired material properties. Furthermore, natural cross-linkers can sometimes result in materials with lower cross-linking density compared to their chemical counterparts, which may affect structural integrity and long-term stability.^63^

The future development of cross-linking methods should focus on combining chemical and natural cross-linkers to optimize performance while minimizing health and environmental risks. Research should also aim at improving processing techniques to reduce costs and enhance the scalability of using natural cross-linkers in industrial applications.

A fundamental distinction between chemical and physical cross-linking lies in the stability of the resulting polymer network. While physically crosslinked PVA relies on hydrogen bonding and crystalline domains, rendering it responsive to environmental conditions, chemically crosslinked PVA maintains its structural integrity even under extreme conditions, such as elevated temperatures and prolonged aqueous exposure.^64^ This exceptional stability makes chemically crosslinked PVA highly suitable for applications requiring long-term durability.^65^ However, the use of chemical cross-linking agents can pose challenges, particularly in biomedical applications, due to potential cytotoxicity. Consequently, optimizing cross-linking conditions not only enhances performance but also minimizes potential health and environmental risks.^66,67^

Despite these challenges, chemically crosslinked PVA has demonstrated significant potential across various industrial and environmental applications. For instance, sulfosuccinic acid (SSA)-crosslinked PVA membranes exhibit excellent ion selectivity and mechanical durability in the treatment of radioactive wastewater.^35,68^ Similarly, citric acid-crosslinked PVA films have emerged as promising candidates for biodegradable packaging materials, offering enhanced mechanical strength and barrier properties that are crucial for food preservation and sustainability initiatives.^69^ These advancements underscore the versatility of chemically crosslinked PVA, highlighting its ability to meet the demands of high-performance engineering applications while supporting environmentally responsible material development.

#### Hybrid Cross-linking Approaches

Hybrid cross-linking combines both physical and chemical cross-linking techniques to achieve a balance between mechanical strength, flexibility, and biocompatibility. This approach is particularly advantageous for applications that require materials with tailored properties, as it combines the elasticity of physically crosslinked networks with the enhanced strength of chemically crosslinked structures. Typically, PVA undergoes an initial physical cross-linking step, such as freeze–thaw cycling, to induce the formation of crystalline regions, followed by chemical cross-linking using agents like CA or genipin to introduce covalent bonds. This dual strategy allows for precise control over cross-linking density and network architecture, resulting in materials with superior mechanical and structural properties.^70,71^

The main advantage of hybrid cross-linking lies in its ability to optimize material performance by leveraging the strengths of both cross-linking methods. Physical cross-links contribute to elasticity and flexibility, while chemical cross-links provide long-term stability and resistance to environmental degradation. Moreover, this method mitigates some of the drawbacks associated with chemical cross-linking, such as potential cytotoxicity, by reducing the required amount of chemical cross-linking agents. As a result, hybrid crosslinked PVA materials exhibit enhanced mechanical properties compared to purely physically cross-linked materials, while maintaining better biocompatibility than fully chemically cross-linked.^64,71^

For example, Nguyen et al. (2023) developed a hybrid PVA scaffold for cartilage repair, integrating physical cross-linking to support cellular interactions and chemical cross-linking to enhance mechanical strength and structural integrity.^72^ The resulting scaffold demonstrated excellent mechanical properties and promoted cell adhesion and proliferation, highlighting its potential for tissue-engineering applications.^64^

The flexibility of hybrid cross-linking has also been explored in drug-delivery systems, where physical cross-links facilitate controlled swelling and drug diffusion, while chemical cross-links maintain structural integrity. A Ribeiro. et al. (2023) reported a hybrid PVA material for sustained release of anticancer drugs, demonstrating controlled drug release kinetics and improved stability,^73^ further emphasizing the importance of hybrid cross-linking in biomedical applications.^71^ These advancements highlight the potential of hybrid cross-linked PVA in developing multifunctional materials suitable for high-performance medical and engineering applications.

In summary, the cross-linking method plays a crucial role in determining the structural and functional properties of PVA-based materials. Physical cross-linking, such as freeze–thaw cycling, forms reversible hydrogen bonds and crystalline domains, providing excellent biocompatibility and responsiveness to environmental conditions without introducing toxic agents. Chemical cross-linking, through covalent bonds formed by agents like glutaraldehyde or citric acid, enhances the material’s mechanical strength, thermal stability, and long-term durability. Hybrid cross-linking, which integrates both physical and chemical mechanisms, offers a balanced strategy to tailor material properties for specific applications. This combined approach enables the development of advanced materials with improved performance and multifunctionality. With ongoing research and refinement, these cross-linking strategies, especially hybrid systems, show strong potential for expanding the use of PVA materials in fields such as biomedicine, environmental engineering, and bioenergy conversion.

### Grafting

Grafting is a modification technique that incorporates additional monomers or polymers onto a polymer backbone chain via covalent bonding. Therefore, grafting is a common pathway that is utilized not only to elevate PVA’s qualities but also to impart novel applications of this material.

Although grafting polymers has been studied since 1940–1950, scientists have only been grafting polymer branches onto PVA in the 1960s in an effort to enhance PVA’s characteristics.^74^ The process typically involves activating the PVA side chains and target polymer chains through chemical approaches to facilitate the covalent bonding between the two components.^75,76^

PVA grafting reactions are classified by grafting mechanisms: grafting “onto” and grafting “from”. Grafting onto implies the attachment of prepolymerized chains (e.g., polysaccharides, proteins) to the reactive PVA backbone. Meanwhile, grafting from means that the PVA polymer backbone, with initiation side functions as a macroinitiator from where side chains are grown.^77^ Figure 3 summarizes the two main methods for synthesizing graft copolymers of PVA. This distinction is fundamental to understanding grafting efficiency, structure–property control, and environmental impacts. In this review, we will provide an in-depth analysis of these methods backed by recent research, as well as several comparison tables.

**Figure 3:**
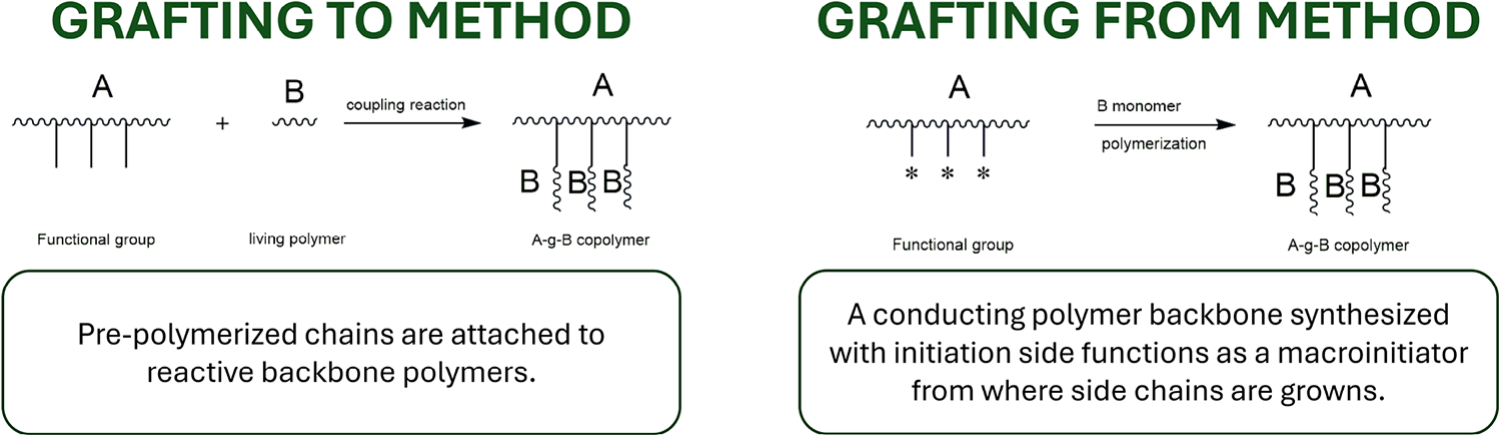
Schematic chart of different PVA grafting methods and examples.

#### Grafting onto PVA

Grafting onto PVA typically involves condensation, esterification, or coupling reactions between the hydroxyl (−OH) groups of PVA and the reactive functional groups of the activators or the grafts themselves. Comparable to chemical cross-linking, the method is to establish permanent covalent bonds, but the aim is to attach polymer chains or reactive molecules onto the backbone of the PVA polymer chain. Although “grafting onto” is most often associated with condensation or coupling reactions, it can also occur through radical recombination when both PVA and the preformed polymer bear active radical sites.

The grafting of PVA involves activators or coupling agents (e.g., CDI, EDC-HCl) promoting esters or amide bond formation, and chemical initiators are frequently used to enhance its efficiency. Moreover, click chemistry was also observed in some PVA studies, made possible through tosylation and azidation of the hydroxyl groups.^78^ After the PVA backbone is modified chemically, grafted chains or molecules branch out from the main polymer backbone, resulting in a comb-like structure. Utilization of chemical activators in grafting often elevates PVA’s interfacial adhesion, mechanical properties,^79^ thermal stability,^80^ and bioactivity, while also enhancing applications of the material in various areas.^10,81–83^ The main advantages of PVA grafting over freestanding polymers are the ability to modify the properties of the final product and the potential for improved performance.^84^

A variety of reactants can be utilized to perform “grafting onto” reactions, with each bringing unique properties. Table 6 illustrates the detailed examples and compares the diverse grafting onto PVA’s backbone in the literature can be found below.

**Table 6:**
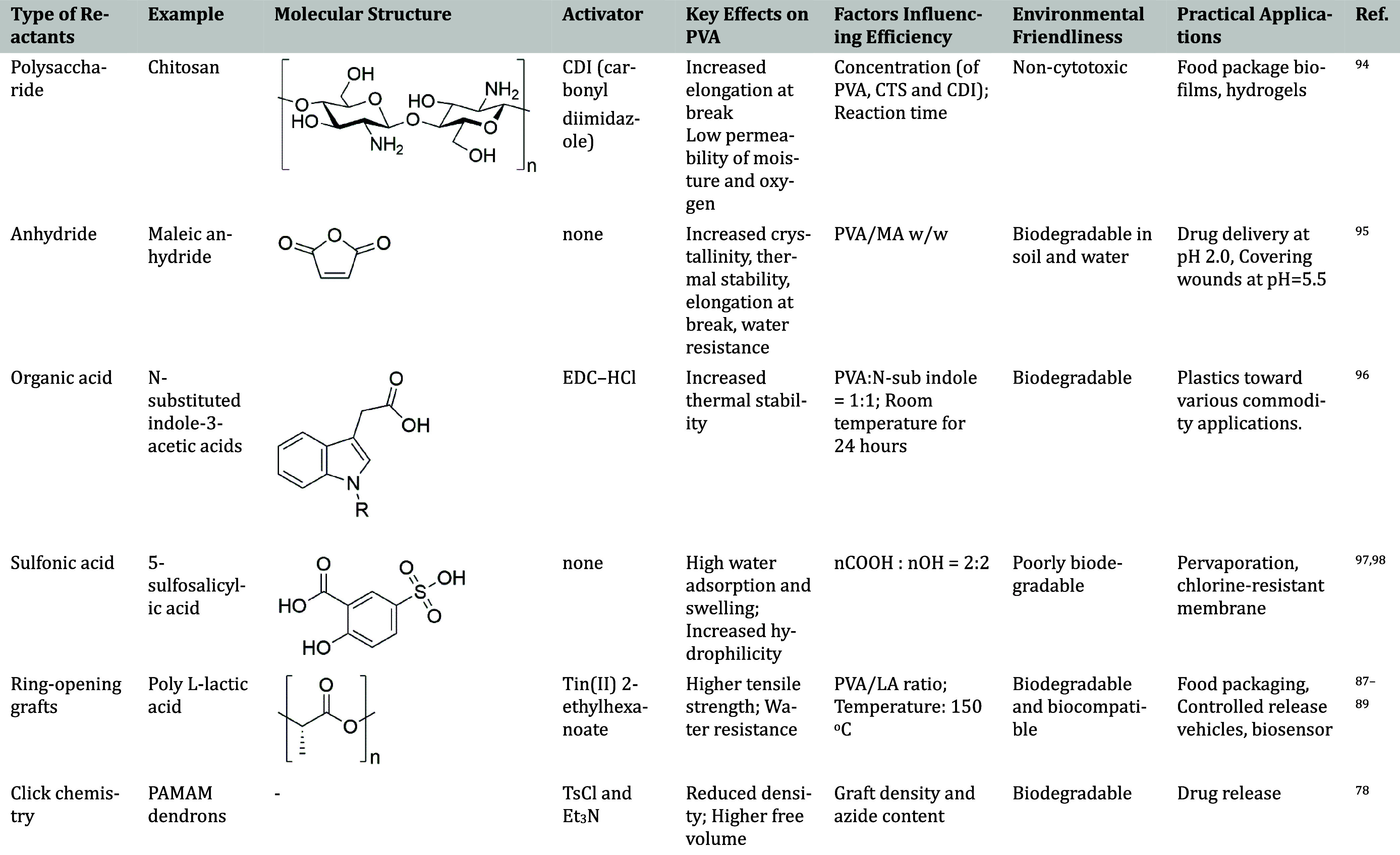
Classification of Covalent Bonding Agents for PVA Grafting^96–98^

It is noteworthy that biopolymers can be easily grafted onto PVA through PVA functionalization by carbonyldiimidazole (CDI). This allows PVA to undergo more modifications without compromising its nontoxicity and biodegradability. Additionally, the grafting of proteins like collagen with PVA fibers has proven to be efficacious, thereby potentially expanding their applications in the field of tissue engineering.^85^

Table 7 contrasts PVA grafted with maleic anhydride (PVA-*g*-MA) at different weight ratios of PVA to MA (PVA/MA) to show how reactant content affects material qualities. This comparison emphasizes how important reaction parameters are in affecting PVA’s mechanical strength, thermal stability, and biodegradability. This study explains the trade-offs between grafting degree and mechanical performance, as excessive reactant led to homopolymerization and overall weaker PVA films.

**Table 7:** Influence of the Monomer-to-PVA Ratio on Material Properties^95^

	PVA/MA = 8:2	PVA/MA = 7:3	PVA/MA = 5:5
Parameter	w/w	w/w	w/w
Grafting efficiency (%)	<80%	∼87%	∼85%
Particle size (nm)	-	120 nm (largest)	60 nm
Dynamic viscosity (cps)	<150 cps	150 cps	100 cps
	Lower	Highest (corresponds to highest grafting)	Lowest (chain scission and gel formation)
XRD	Highest intensity (sharp peak at 20.18°)	High intensity (sharp peak at 19.53°)	Lowest intensity (sharp peak at 18.83°)
Swelling ratio (%)	200%	150%	450% (1 day)
			550% (7 days)
Tensile strength (MPa)	17.5 MPa	15 MPa	1 MPa
Elongation at break (%)	850%	800%	400%
Thermal stability	Moderate	Highest	Lowest
Biodegradability (%)	48%	35%	55%

Most grafting onto approaches exploit the reactive hydroxyl groups of the PVA backbone to form stable ester or ether linkages with preformed polymers or small reactive molecules. Such condensation-based mechanisms provide greater control over grafting sites and minimize homopolymerization, leading to well-defined copolymers with predictable structures.^86^ In contrast, free-radical-based approaches, as discussed in the next section, enable a broader range of vinyl monomers but rely on hydrogen abstraction and propagation steps rather than direct hydroxyl groups participation.

In general, grafting onto approaches produces PVA derivatives with enhanced mechanical strength, controlled crystallinity, and adjustable hydrophilicity depending on the grafted molecule. The use of mild activators such as CDI or EDC–HCl enables efficient bonding without compromising PVA’s biodegradability, while ring-opening grafts with lactide or related monomers further improve tensile and thermal properties.^87–89^ These findings highlight the effectiveness of grafting strategies for producing biocompatible and environmentally benign PVA copolymers.

#### Grafting from PVA

In contrast, “grafting from” uses the PVA chain itself as a macroinitiator. Here, active sites are generated directly on PVA, either chemically or physically, from which new polymer chains grow in situ. Compared to the grafting to approach, where larger polymer chains must diffuse to the reactive sites, which may be protected by the already-grafted polymer chains, the grafting from method is thought to produce a higher grafting efficiency because it reduces the steric hindrance of diffusing small monomers.^90^

Grafting onto strategy utilizes the reactive hydroxyl groups of the PVA backbone, facilitating easier control of products, while free-radical grafting offers a greater range of grafted materials, and their resistance to impurities.^86^ Free radicals are created by the initiators and result in hydrogen abstraction at the methine carbons of the PVOH followed by propagation of monomer.^91,92^ This method allows for modification of the PVA backbone without necessarily causing reactions in the hydroxyl groups. Graft polymerization of vinyl monomers, for instance, has garnered a lot of attention due to the desirable features it imparts, including protein adsorption, ion exchange, chelation, and biocompatibility.^93^

The ratio of PVA to grafting monomers and the processing parameters, including temperature, reaction time, and solvent composition, critically determine the structure and performance of the grafted PVA copolymers. In free-radical grafting systems, elevated temperatures can accelerate initiator decomposition, thereby generating more free radicals and active sites on the PVA backbone, which initially increase the grafting degree. However, when the temperature exceeds the optimal range, premature chain termination and chain-transfer reactions dominate, resulting in decreased grafting efficiency.^99^ Excessive thermal energy can also trigger backbiting reactions at the propagating radical sites, shortening side chains and lowering molecular weight.^100^

The kinetics of grafting may depend differently on reaction time. In some cases, the reaction rate is governed primarily by the concentration of initiators, reactants, and PVA, independent of time. These factors influenced the quantity of active sites on the backbone of PVA, which is directly related to how a polymer reacts with initiators and reactants. Consequently, a graft yield peak will be seen as the feed concentration rises, followed by a sharp decline. This implies that at some time, homopolymerization will be more preferred to copolymerization.^101^ Whereas other systems display a steady increase in grafting degree with longer reaction durations due to diffusion-controlled propagation.^102^ For surface modification grafting, excessive reaction time may lead to the accumulation of unreacted PVA or grafting agents, deteriorating surface uniformity.^103^

Furthermore, the monomer-to-PVA ratio influences the balance between the grafting degree and yield. Although increasing monomer concentration may promote graft formation, it often reduces grafting yield because of bond scission and formation of multiple shorter chains relative to the original backbone.^104^ These parameters collectively determine the molecular architecture, mechanical stability, and surface characteristics of PVA-graft copolymers, highlighting the importance of optimizing the reaction conditions for each polymerization system.

Depending on the initiation mode, the reaction can proceed via radical, controlled radical, ring-opening, irradiation, or microwave-assisted polymerization. Below, we will discuss each polymerization approach in detail.

##### Free-Radical Polymerization

With this process, initiators produce free radicals, which react with the monomer to cause the polymeric chain to develop.^105^ Peroxides, redox initiators, azo groups, and photoinitiators are a few examples of various types of initiators for free-radical systems.

Potassium persulfate (KPS) is the most utilized initiator because of its inexpensiveness, low decomposition temperature, and strong water solubility. Grafting various vinyl monomers onto PVA chains has been accomplished using KPS. Chouhan et al. (2015) grafted acrylonitrile (AN)—a thermoplastic material with high thermal stability onto PVA and continued to incorporate zinc oxide to create a UV-blocking, antibacterial composite.^106^ Similarly, acrylic acid (AA) has been effectively grafted onto PVA by He and Zhang (2018) to provide a water-soluble binder material for the Si anode in lithium-ion batteries.^107^ Yang et al. (2012) demonstrated how the quantity of initiator KPS affected grafting efficacy using PVA-*g*-NIPAM (*N*-isopropylacrylamide) films. The article concluded that at a particular initiator concentration, the equilibrium of two opposing polymerization processes—graft copolymerization onto the PVA main chains and homopolymerization of monomers—would be achieved.^108^

Other initiators that have been successfully used in the free-radical grafting of PVA include heavy metal salts (e.g., ceric ammonium sulfate-CAS,^109^ ceric ammonium nitrate-CAN^110^), water-insoluble azo initiators (e.g., azobis­(isobutyronitrile)-AIBN,^111^ or hydrogen peroxide.^112^ A study employing CAN as an initiator demonstrates that the oxidizing behavior of initiators is also influenced by the pH environment of the aqueous solution pH environment. Furthermore, the type of acid used has an impact in addition to the acid concentration.^113^

Free-radical grafting-from methods are valued for their versatility and simplicity, accommodating a wide range of vinyl monomers and initiators, such as KPS, CAN, or AIBN under relatively mild conditions. These reactions are experimentally straightforward, require no prefunctionalization, and are compatible with diverse aqueous or solvent systems, making them scalable for industrial use. Some desirable properties added into PVA material include: tunable hydrophilic/hydrophobic balance, improved thermal stability, and enhanced UV resistance.

However, free-radical grafting offers limited molecular control, often producing broad dispersity and undesired homopolymer formation. Side reactions such as backbone scission, cross-linking, and oxidation may also occur, complicating purification and affecting mechanical properties.^114,115^ Despite these drawbacks, its high reactivity, cost-effectiveness, and adaptability sustain its dominance in the chemical modification of PVA for hydrogels, membranes, and coatings.

##### Controlled/Living Radical Polymerization

To overcome the limited molecular control inherent to conventional free-radical grafting, controlled/living radical polymerization (CRP) techniques such as photoinduced electron transfer–reversible addition–fragmentation chain transfer (PET-RAFT) and surface-initiated atom transfer radical polymerization (SI-ATRP) have been successfully adapted for PVA modification. In these approaches, polymerization proceeds through a reversible activation–deactivation mechanism, which minimizes chain termination and enables precise control over molecular weight, graft density, and chain architecture.

In the SI-ATRP method, halogenated PVA derivatives act as macroinitiators, while copper­(I) bromide complexes with ligands such as *N*,*N*,*N*′,*N*′,*N*″-pentamethyldiethylenetriamine (PMDETA) mediate the redox equilibrium between dormant and active species.^116^ Grafting glycidyl methacrylate (GMA) onto PVA nanofibers provided reactive epoxy functionalities capable of covalently immobilizing α-amylase, demonstrating the effectiveness of “grafting onto” in introducing bioactive surfaces without compromising the mechanical integrity of the PVA matrix. The modified PVA nanofibers exhibited enhanced enzyme loading and retained structural stability, confirming that surface grafting can successfully combine the chemical reactivity required for biomolecule attachment with the inherent flexibility and robustness of PVA.

Alternatively, PET-RAFT polymerization offers a catalyst-free, light-driven route in which photoexcited electron donors activate chain transfer agents attached to PVA or its functionalized precursors. The technique, developed by Boyer et al., provides a green, versatile route for synthesizing precision polymers under mild conditions. It offers broad light-source compatibility, minimal waste generation, oxygen tolerance, and spatial-temporal control, making it well-suited for sustainable polymer fabrication.

For example, near-infrared (NIR) light–induced PET-RAFT polymerization was employed to graft poly­(carboxybetaine methacrylate) (pCBMA) chains onto PVA hydrogel surfaces, forming highly hydrated, zwitterionic brush layers that markedly improved antifouling resistance and biocompatibility.^117^ The resulting PVA-*g*-pCBMA hydrogels exhibited significantly enhanced surface hydrophilicity and resistance to protein adsorption while maintaining excellent cytocompatibility with human fibroblasts. The study demonstrated that light-mediated “grafting onto” via PET-RAFT is an effective strategy for fabricating functional antifouling PVA materials suitable for biomedical applications.

While these controlled polymerization strategies offer superior precision, they also face practical limitations. ATRP systems require metal catalysts whose appearance may constrain applications unless stringent removal is implemented.^118^ PET-RAFT systems, though catalyst-free, can exhibit photochemical vulnerabilities such as photocatalyst photobleaching and light-penetration.^119^ Despite these challenges, both PET-RAFT and SI-ATRP represent significant advances toward well-defined, functional PVA-graft copolymers, combining the architectural control of living polymerization with the biocompatibility of PVA.

##### Irradiation and Microwave-Induced Polymerization

Traditional approaches often rely on chemical initiators and catalysts; however, these can introduce impurities, require stringent conditions, and pose environmental concerns. As such, alternative methods that utilize physical means of initiation, particularly radiation-based techniques, have gained increasing attention for their precision, cleanliness, and ability to operate under milder conditions.

It is also possible to create active sites, or free radicals, on a polymeric surface using ultraviolet (UV), γ (e.g., 60Co), and electron beam (EB) radiation. These can then react with vinyl monomers to create a graft copolymer. Initiator, catalyst, and cross-linker are not necessary for the radiation processing method; nevertheless, it does require high doses of ionizing radiation (tens or hundreds of kilogray (kGy).^120^ Moreover, this technology has demonstrated the capacity to streamline the entire treatment procedure and to decrease production costs.

The primary benefits of radiation-induced grafting compared to traditional chemical grafting include: the reaction can occur across a broad temperature spectrum; grafting can be performed in diverse monomer states (including bulk, solution, emulsion, and solid); and the resultant material is the absence of residual initiators or catalysts.^121^

Similar to the use of chemical activators, the weight ratio of reactants to PVA, pH, solvent system, reaction duration, and temperature all have an impact on the grafting process. The grafting process is also significantly affected by radiation exposure. Studies have also demonstrated that, at specific concentrations, mineral acids can improve the grafting process.^122^

Irradiation-induced grafting can be performed by two major methods: a mutual or simultaneous technique or a preirradiation technique.^123^ Typically, grafting is performed in solution or emulsion, where the reaction medium is usually water with a small amount of surfactant.^124^ In the first approach, the polymeric material is subjected to irradiation while immersed in a monomer solution (or pure monomer). This straightforward technique is frequently used to modify the surface of polymeric materials and works well with radiation-sensitive substrates. However, because radicals other than those in the polymeric backbone can consume monomers, a variety of side reactions can also occur, limiting the extent of grafting.^125^

Preirradiation is often preferred because irradiation is conducted in vacuum or in an inert atmosphere; therefore, less prone to produce side reactions. Typically, elevated dose rates are necessary to achieve a greater concentration of radicals, and normally, some heating is required to initiate the reaction. Depending on the monomer reactivity, an activated temperature is generally needed. In a study by Tian et al. (2017), polybutadiene rubber latex was preirradiated before being successfully reacted with PVA, resulting in a rubber material that was both vulcanized and chemically modified, resulting in enhanced hydrophilicity.^126^ This method’s primary benefits over chemical activators are its repeatability, simplicity, and cleanliness. Furthermore, heat-sensitive reactants can benefit from the reaction because it often does not necessitate extreme temperatures.^121,127^

Graft copolymerization with the assistance of microwaves is more appealing and environmentally friendly. As evidenced by the high product yield, this type of grafting reduces the need for toxic solvents, shortens the reaction time, and produces fewer byproducts. Through selective excitation, microwave radiation (MW radiation) breaks down polar bonds, producing free radicals on the polymeric backbone.^128^ Using only KPS as an initiator, a PVA-*g*-(Starch-*g*-PMA) matrix was created under microwave irradiation with a very high yield; the material was found to have increased gas-separation capabilities.^129^ Likewise, using ammonium persulfate (APS) as the redox initiator, polyacrylamide was effectively grafted onto a blend of PVA and fenugreek seed mucilage with up to 96% graft efficiency utilizing 30-s microwave irradiation.^130^

In summary, radiation-induced and microwave-assisted graft copolymerization methods offer efficient, flexible, and environmentally benign alternatives to conventional chemical grafting. These approaches not only eliminate or reduce the need for toxic initiators and high temperatures but also enable precise control over the reaction, resulting in improved material properties and high grafting efficiency. Continued research and optimization of these techniques hold promise for the development of advanced functional materials in fields such as packaging, biomedical, and environmental uses.

In general, studies investigating this method of PVA grafting have shown notable improvements in the thermal stability of the PVA backbone.^124,130^ Interestingly, the resulting copolymer characteristics vary widely depending on the grafted component: hydrophilic grafts enhance swelling and water uptake,^128^ hydrophobic moieties impart moisture resistance,^125^ and amphiphilic structures offer balanced interfacial behavior.^131^ Moreover, simultaneous grafting of multiple monomers has been explored to fine-tune PVA’s structural and surface properties, creating extensive opportunities for tailoring materials for specific applications.

### Functionalization

#### Esterification

Esterification of PVA refers to the reaction between the hydroxyl groups (−OH) of PVA and esterifying agents, such as carboxylic acids, acid anhydrides, or acid chlorides, resulting in the formation of ester linkages (−COO−) along the polymer backbone. Figure 4 shows the general reaction scheme of the PVA esterification.

**Figure 4:**
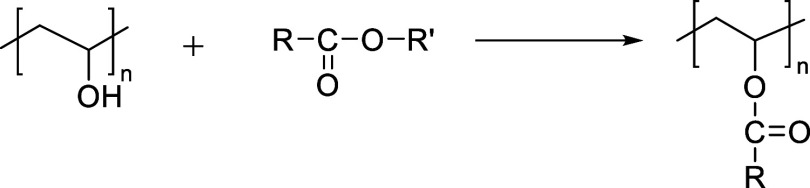
General reaction scheme of PVA esterification.

Among these, carboxylic acids are commonly used due to their availability, biocompatibility, and compatibility with green chemistry. Depending on the acid type, aliphatic, aromatic, or multifunctional, the modified PVA may exhibit properties such as pH responsiveness or proton conductivity.

The esterification of PVA with carboxylic acids typically follows a nucleophilic acyl substitution mechanism, in which hydroxyl groups (−OH) on the PVA backbone react with the carboxyl groups (−COOH) of organic acids to form ester linkages (−COO−), along with the release of water or another byproduct depending on the reagent used. The reaction efficiency depends heavily on the acid structure, the number of carboxyl groups, the reaction environment (thermal, catalytic, or solvent-based), and the reactivity of the acid.^50–53,131^

One widely used approach is thermal esterification, especially with di- and tricarboxylic acids. This method generally occurs at elevated temperatures (90–120 °C) without added catalysts and promotes the formation of ester bonds by removing water through evaporation.^50,52^ For example, maleic acid was shown to react with PVA at 120 °C for 60 min, forming a covalently cross-linked network and significantly reducing water swelling from 230 to 30%.^50^ In solid-phase esterification, the reaction proceeds without the use of solvents or catalysts, primarily driven by the proximity of molecules during the drying phase. This green process has been applied to create materials with high mechanical strength, self-healing ability, and water resistance.^39,51^

When higher reactivity is required or multifunctional networks are desired, acid-catalyzed esterification is employed. In several systems, hydrochloric acid (HCl) at pH ≈ 1 is used to catalyze the esterification at mild temperatures.^49^ These conditions allow for partial preservation of functional groups (−COOH, −NH_2_), contributing to pH-sensitive swelling and controlled drug release.^49^

Characterization techniques used to confirm esterification include Fourier Transform Infrared Spectroscopy (FTIR) (peak of the ester carbonyl groups), Nuclear Magnetic Resonance (NMR) (^1^H, ^13^C) for chemical structure, and Differential Scanning Calorimetry/X-ray Diffraction (DSC/XRD) to evaluate changes in thermal behavior and crystallinity. Additional tests such as water swelling, mechanical tensile testing, and Scanning Electron Microscopy (SEM) imaging are used to assess final material performance.^49–53,131^

The esterification of PVA with various carboxylic acids significantly enhances its material properties, enabling diverse practical applications. Linear aliphatic acids such as succinic and glutaric acid yield esterified PVA with balanced crystallinity and mechanical flexibility, suitable for biodegradable films, packaging materials, and hydrogels.^49,54^ Highly reactive acids like malonic and maleic acid promote rapid esterification and form densely cross-linked structures, applicable in moisture barrier films and fast-curing adhesives.^50,51^

The incorporation of aromatic acids improves rigidity and thermal stability, making them useful in technical coatings and conductive membranes.^55,131,132^ Additionally, specialty acids such as 1,2,3,4-butanetetracarboxylic acid (BTCA) and sulfosuccinic acid create highly functional networks with enhanced dimensional stability, wrinkle resistance, and ionic conductivity, used in textile finishing and membrane technologies.^56,133^

A green biobased modifier, l-glutamic acid (l-Glu), has been used to introduce functional groups into the PVA backbone. In a 2025 study, l-Glu-modified PVA was combined with poly­(ethylene glycol) (PEG), which served both as a porogen and a plasticizer, enhancing the surface area and adsorption efficiency. l-Glu provided specific adsorption sites, where the amine group (−NH_2_) in its structure exhibited a much higher affinity for Cu­(II) ions than the hydroxyl group (−OH), resulting in superior adsorption capacity along with excellent safety and biocompatibility. The Cu­(II) adsorption capacity increased from 31.8 to 51.1 mg/g, with efficient desorption (81.9% in 2 M HNO_3_) and reduced crystallinity, highlighting the potential of l-Glu/PEG-modified PVA as a sustainable porous membrane for heavy metal removal.^134^

The esterification of PVA offers tailored properties for various applications, but each type of acid also presents specific limitations. Linear aliphatic acids provide flexibility and biodegradability but may result in lower cross-linking density and water resistance.^49,54^ Reactive short-chain acids enable fast cross-linking; however, they may reduce elasticity or leave residual acidity, affecting biocompatibility.^50,51^ Aromatic acids enhance thermal stability and rigidity, yet may increase brittleness or reduce degradability.^55,131,135^ Polyfunctional acids form dense networks beneficial for biomedical use, though excessive cross-linking can compromise flexibility or lead to hydrolytic instability over time.^52,53^ Specialty acids introduce advanced functionalities like ionic conductivity or wrinkle resistance but may involve more complex synthesis routes or higher cost.^56,133^

Esterification of PVA using anhydrides is a highly efficient approach, which allows ester bond formation under mild or solvent-free conditions. Depending on the structural nature of the anhydride, whether aliphatic, aromatic, or dianhydride, the resulting PVA network may exhibit varying levels of stiffness, flexibility, or hydrophilicity. Reaction efficiency is further influenced by factors such as the processing temperature, reactant ratio, and steric accessibility.

The esterification of PVA with anhydrides generally follows a nucleophilic ring-opening mechanism in which the hydroxyl groups of PVA attack the electrophilic carbonyl carbon in the anhydride ring, leading to the formation of ester bonds. This reaction occurs efficiently under solvent-free or melt-state conditions and does not typically require a catalyst. In monoanhydride systems, such as those involving aliphatic or aromatic anhydrides, the reaction is often conducted by mixing the anhydride with partially dried or film-cast PVA, followed by thermal treatment at temperatures ranging from 100 to 150 °C for 30 min to 2 h. The molar ratio of hydroxyl to anhydride is generally adjusted around 1:1 or 2:1 to optimize the degree of substitution.^102,135–138^

The cross-linking of PVA using aromatic dianhydrides such as 3,3′,4,4′-benzophenone tetracarboxylic dianhydride (BTDA), 4,4′-oxidiphthalic anhydride (ODPA), and pyromellitic dianhydride (PMDA) illustrates how minor variations in molecular structure can significantly influence membrane properties. All three dianhydrides reduce hydroxyl content and enhance cross-linking, yet they differ in their effects on hydrophilicity, swelling behavior, and pervaporation performance. PMDA yields the highest degree of cross-linking and swelling resistance, followed by ODPA and BTDA, resulting in the highest separation factor for PVA/PMDA membranes, while all three maintain comparable flux due to similar increases in free volume.^139^

Table 8 summarizes the reaction mechanisms and structural features of various anhydrides used in the PVA esterification.

**Table 8:**
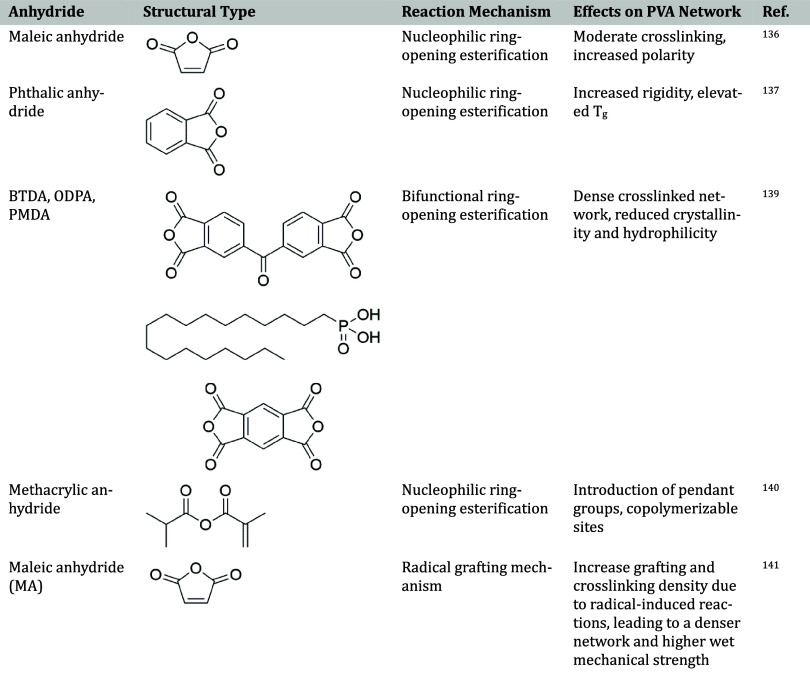
Reaction Mechanism and Structural Features of Anhydrides Used in PVA Esterification^141^

Nucleophilic ring-opening esterification is a general strategy in which hydroxyl groups of PVA act as nucleophiles to attack electrophilic carbonyl carbons of cyclic anhydrides or lactones. This leads to ring-opening and the formation of ester linkages along with pendant carboxylic acid groups. Such reactions enhance the functional density of PVA and can provide sites for further cross-linking.

In some cases, base-catalyzed nucleophilic esterification is employed to accelerate the reaction rate and improve the selectivity for ester bond formation. Basic catalysts activate hydroxyl groups by deprotonation to generate alkoxide ions, which are stronger nucleophiles. These alkoxides then attack the electrophilic carbonyl centers of anhydrides or acyl compounds, resulting in efficient ring-opening and esterification, even under mild thermal conditions.

While monofunctional cyclic compounds can effectively introduce ester linkages and pendant functional groups onto PVA chains, they typically result in linear or lightly branched structures. To further increase the degree of cross-linking and tailor the network architecture, bifunctional ring-opening esterification has been employed. In this approach, bifunctional cyclic monomers containing two reactive sites can simultaneously react with multiple hydroxyl groups of PVA, leading to the formation of three-dimensional cross-linked or grafted networks with enhanced structural stability and tunable physicochemical properties.

In addition to these nucleophilic pathways, free-radical grafting reactions have also been utilized to introduce anhydride functionalities onto PVA. In this mechanism, radicals generated by initiators such as potassium persulfate, benzophenone, or benzoin ethyl ether abstract hydrogen atoms from hydroxyl groups of PVA, producing macroradicals along the polymer backbone.

These macroradicals subsequently add to the double bonds of maleic or succinic anhydride, forming C–C graft linkages and occasionally secondary ester bonds. Such radical-induced grafting provides an alternative route for PVA functionalization under relatively mild thermal or photoirradiation conditions.

Anhydrides are highly effective in modifying PVA by reducing free hydroxyl groups, disrupting hydrogen bonding, and increasing cross-link density, which improves water resistance, dimensional stability, and thermal properties such as elevated *T*_g_ in aromatic systems.^135–137^ The outcome depends on the anhydride’s structure: aliphatic types provide flexible networks with lower *T*_g_, while aromatic and dianhydrides form rigid, hydrophobic matrices with reduced crystallinity.^135,139^ Methacrylic anhydride also enables grafting of vinyl groups, opening pathways for further functionalization.^140^

The esterification of PVA with acid chlorides follows a nucleophilic acyl substitution mechanism in which hydroxyl groups on the PVA backbone attack the carbonyl carbon of the acid chloride, forming ester linkages. The esterification process of PVA using acid chlorides is illustrated in Figure 5.

**Figure 5:**
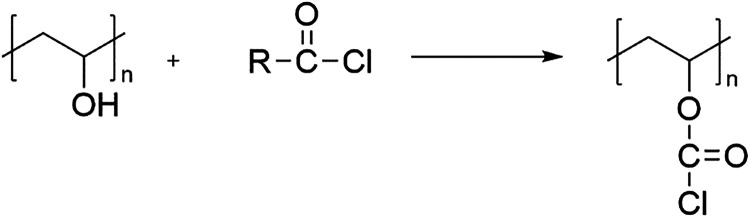
Esterification of PVA using acid chlorides.

In several studies, the reaction was carried out by treating PVA films or solutions with acid chlorides in organic solvents (tetrahydrofuran (THF), *N*,*N*-dimethylformamide (DMF), at temperatures ranging from 25 to 80 °C, and reaction times varying from minutes to several hours, depending on the reagent and molar ratio used.^140,142–145^ Aromatic acid chlorides tend to form surface-bound ester layers that enhance hydrophobicity and reduce surface energy, while long-chain derivatives like lauroyl chloride improve antifriction performance and reduce surface adhesion.^144,145^

Esterification of PVA using anhydrides and acid chlorides is an effective strategy for polymer modification, enhancing properties such as hydrophobicity, thermal stability, mechanical strength, and surface functionality. Anhydrides, particularly aromatic dianhydrides, are widely applied in the fabrication of separation membranes and thermally stable materials, while acid chlorides enable flexible surface modification, suitable for antifouling coatings, low-friction interfaces, and functional packaging systems.^102,136–140,142–145^

However, excessive cross-linking from dianhydrides may lead to brittleness if not well controlled, and acid chlorides generate HCl byproducts, requiring strictly anhydrous conditions and careful safety management. Environmental considerations, such as the chemical origin, solvent usage, and byproduct handling, should be taken into account when selecting suitable esterification reagents.

In summary, esterification offers a versatile approach to modify PVA, with each esterifying agent type providing unique benefits. The choice of reagent should balance performance needs with processing safety and environmental considerations.

#### Acetalization

Acetalization of PVA refers to the reaction of the hydroxyl groups (−OH) of PVA with aldehydes to form acetal linkages (−O–CH–O−) either within or between polymer chains. Depending on the type of aldehyde (aromatic, aliphatic, or heterocyclic), the modified PVA can exhibit a higher *T*_g_, improved thermal stability, reduced water solubility, or enhanced biocompatibility. Factors such as the aldehyde/OH molar ratio, pH, reaction time, and temperature affect the reaction efficiency. The general acetalization mechanism of PVA is depicted in Figure 6.

**Figure 6:**
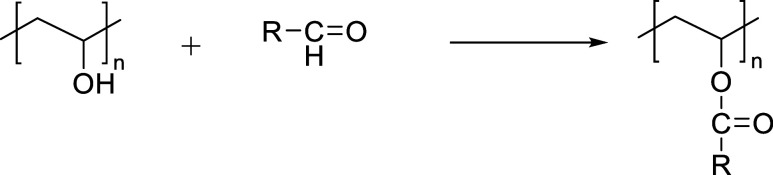
General reaction scheme of PVA acetalization.

Various aldehydes have been used for PVA acetalization, each imparting distinct properties. Aromatic aldehydes such as 1-naphthaldehyde and 9-anthraldehyde improve thermal stability and increase *T*_g_, by their rigid ring structures, while reducing chain flexibility and crystallinity.^146^ Aliphatic aldehydes like formaldehyde, hexanal, heptaldehyde, octyl aldehyde, and 10-undecenal enhance flexibility and hydrophobicity.^147,148^ Certain specialized compounds such as 9-ethyl-3-carbazolecarboxaldehyde contain heteroatoms or electron-donating/withdrawing groups, giving rise to unique electronic, luminescent, or biological properties.^146^ These aldehydes may originate from petrochemical, biobased, or synthetic sources, depending on their structure and intended applications.

Depending on the structure of the acetalization agent, modified PVA can be used in packaging, water-resistant coatings, flexible adhesives, biomedical materials, conductive polymers, and smart responsive films. The choice of agent allows for control over hydrophobicity, flexibility, thermal stability, UV resistance, and surface reactivity. Representative applications and corresponding agents are summarized in Table 9 below.

**Table 9:**
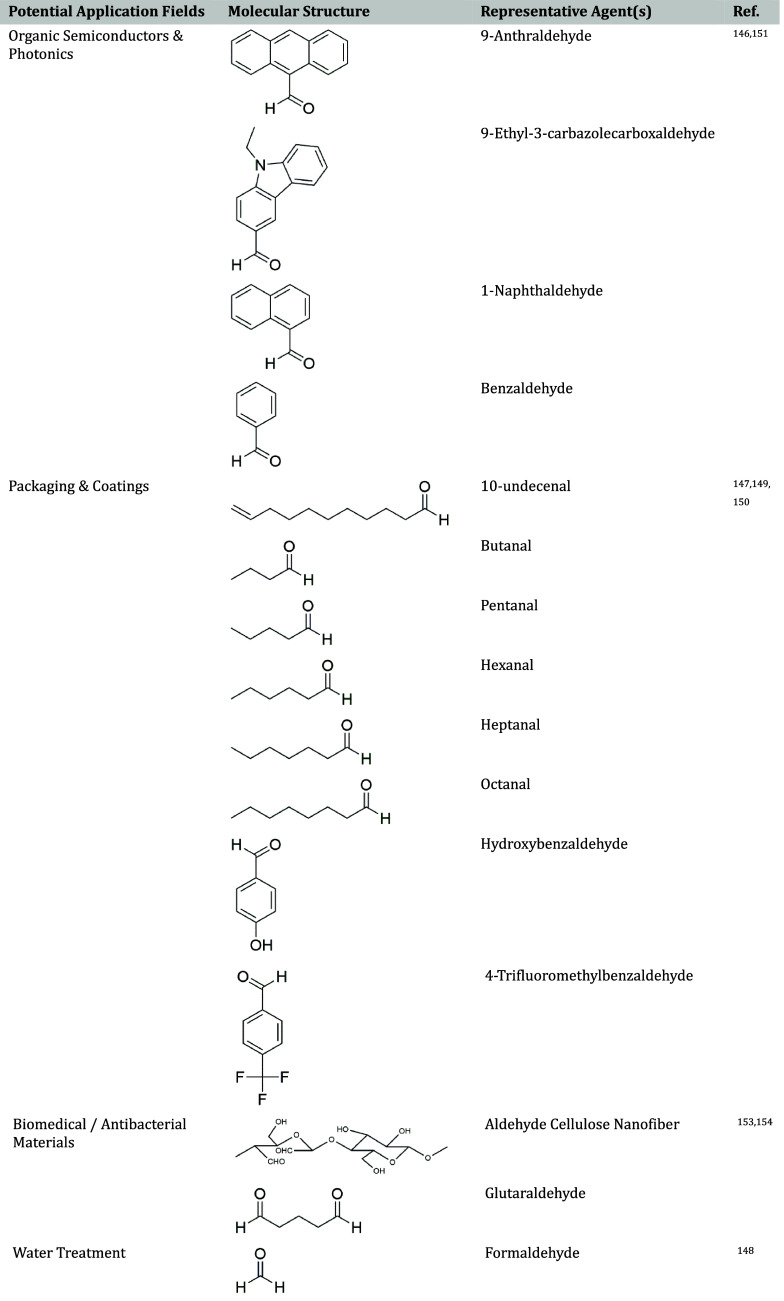
Classification of Acetalization Agents by Application Fields

The acetalization of PVA enhances its thermal and mechanical properties by introducing bulky, rigid, or aromatic aldehyde groups that restrict chain mobility and increase *T*_g_.^146,147,149,150^ This method also enables the tuning of surface properties such as water resistance and hydrophobicity through the incorporation of long-chain or branched aldehydes,^147,149,151–154^ thereby reducing swelling, lowering water solubility, and enhancing barrier performance, which is desirable for packaging, coatings, and membranes.

Some derivatives also offer UV stability or optical activity depending on the chromophore present.^152^ Acetalization provides an effective means to tailor the structural and functional characteristics of PVA-based materials.^147,150,152,155^ By introducing acetal groups of tunable type, size, and content, this reaction modifies the chain polarity, packing density, and intermolecular interactions in a controllable manner. Varying the acetal group length and degree of substitution disrupts crystallinity, reduces hydrogen bonding, and introduces internal plasticization, thereby lowering *T*_g_ and enhancing chain mobility. These molecular-level adjustments enable precise control over solubility, film-forming behavior, and compatibility with other polymers or functional additives, expanding applications to fields such as flexible electronics, drug-delivery systems, smart coatings, and ion-conductive membranes.^150–152,156^

In addition to the acetalization of PVA with aldehydes, the ketalization of PVA with ketones, particularly sustainable, biobased ketones, has attracted significant attention in sustainable polymer materials research. In a recent study, Su et al. (2021) successfully performed PVA ketalization with sustainable ketones, opening new pathways for developing eco-friendly polymer materials.^156^ The general reaction scheme of PVA ketalization using ketones is illustrated in Figure 7.

**Figure 7:**
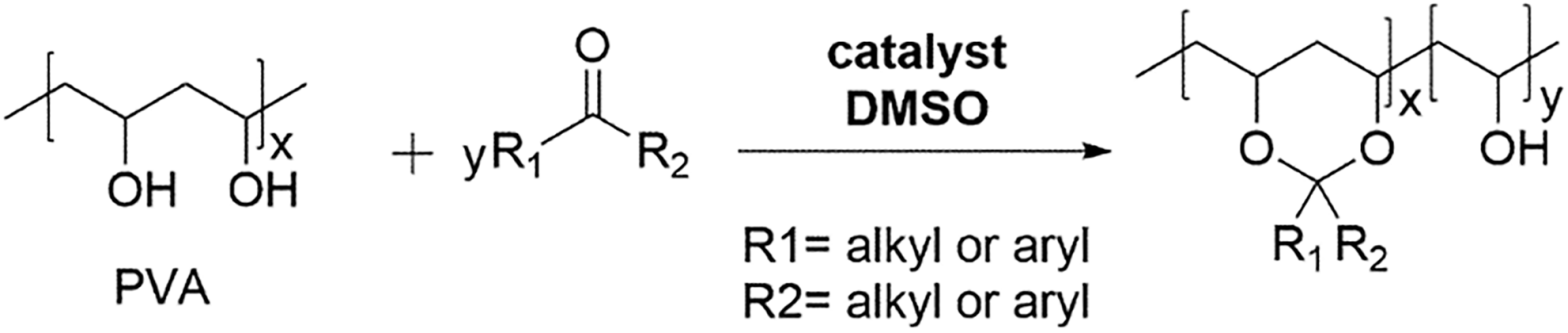
General reaction scheme of PVA ketalization using ketones.

This process, catalyzed by *para*-Toluenesulfonic acid (*p*-TSA) or HCl at 40–60 °C, forms six-membered ketal rings that significantly enhance PVA’s properties. The modified material shows improved thermal stability (*T*_g_ up to 127 °C) and reduced hydrophilicity, while maintaining biodegradability.

The structure and size of the ketones used in the ketalization of PVA have a pronounced influence on the resulting polymer properties. Small aliphatic ketones, such as acetone or butanone, exhibit high reactivity and lead to high degrees of conversion (up to 69.2%), producing uniform poly­(vinyl ketal)­s with moderate increases in *T*_g_ (78–127 °C) and tunable flexibility. In contrast, bulky or biobased ketones such as raspberry ketone, zingerone, or d-dihydrocarvone introduce larger substituents containing aromatic or polar functional groups. These moieties enhance intermolecular interactions, resulting in higher *T*_g_ values (up to 138 °C in the case of furfural acetalization) and improved rigidity, but also reduced chain mobility. These sustainable PVA ketals serve as eco-friendly alternatives to conventional plastics, combining enhanced performance with controlled degradability to meet the rising demand for green materials across various industries.

In summary, acetalization is an effective method to enhance thermal, mechanical, and moisture-resistant properties of PVA, while enabling the tuning of material characteristics through appropriate aldehyde selection. However, the process often requires strong acid catalysts and environmentally harmful solvents, significantly reducing the biodegradability of PVA.

#### Other Methods

The urethanation of PVA primarily proceeds through the nucleophilic addition of hydroxyl groups (−OH) to isocyanate groups (−NCO), forming urethane linkages (−NH–CO–O−). This reaction can occur in various media including solution, bulk, or interfacial systems. Urethanation effectively enhances the physical and chemical properties of PVA, with the final material performance depending on the isocyanate type, reaction conditions, and reactant ratios. As a result, urethanated materials are suitable for applications in packaging, biomedical engineering, and functional materials. Figure 8 presents the general pathway of PVA urethanation.

**Figure 8:**
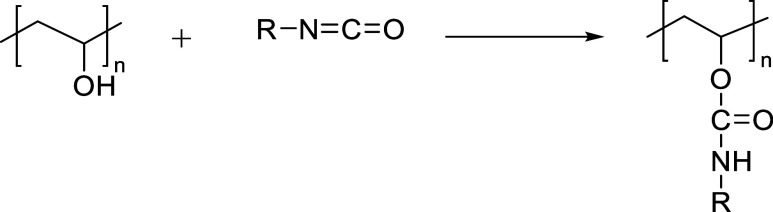
General reaction scheme of PVA urethanation.

When diisocyanates are employed, the urethanation of PVA primarily results in network formation via chemical cross-linking rather than simple chain modification. Therefore, reactions involving diisocyanates are discussed in Challenges and Prospects of PVA Cross-linking Strategies section (*Cross-linking with Diisocyanate*) under the chemical cross-linking category.

Following urethanation, further functionalization, particularly sulfonation, is often employed to meet ionic or electrochemical application needs. Sulfonation of PVA is the process of introducing sulfonic (−SO_3_H) groups onto the PVA backbone; the resulting properties depend on the sulfonating agent, reaction conditions, and degree of substitution. This can be achieved via esterification, Michael addition, or free-radical copolymerization, with reaction conditions such as temperature, catalyst, and sulfonation degree critically influencing the material’s final properties.

The sulfonation of PVA can proceed through various chemical pathways depending on the sulfonating agent used. Commonly, esterification involves sulfonic acid-containing compounds forming ester bonds with PVA’s hydroxyl groups, often aided by cross-linkers like GA under mild to moderate thermal conditions.^157–160^ Michael addition is another approach, using vinylsulfonate monomers such as sodium vinylsulfonate in alkaline media,^161^ while free-radical copolymerization—typically with AMPS or styrenesulfonate derivatives, is initiated by radical sources like benzoyl peroxide.^162^ Advanced methods include click chemistry-based strategies that combine esterification and covalent grafting for high efficiency.^160^ Inorganic sulfonating agents such as sulfated zirconia or sulfonated graphene oxide may also be used, primarily interacting with PVA through hydrogen bonding or physical adsorption.^159^ Chemical modification of PVA via urethanation and sulfonation imparts distinct functional groups that broaden its application scope. Urethanation with isocyanates improves the thermal and mechanical properties for use in films, scaffolds, and packaging. In contrast, sulfonation introduces sulfonic acid groups, enhancing the ionic conductivity and hydrophilicity for membranes and conductive films. Representative agents and applications are compared in Table 10.

**Table 10:**
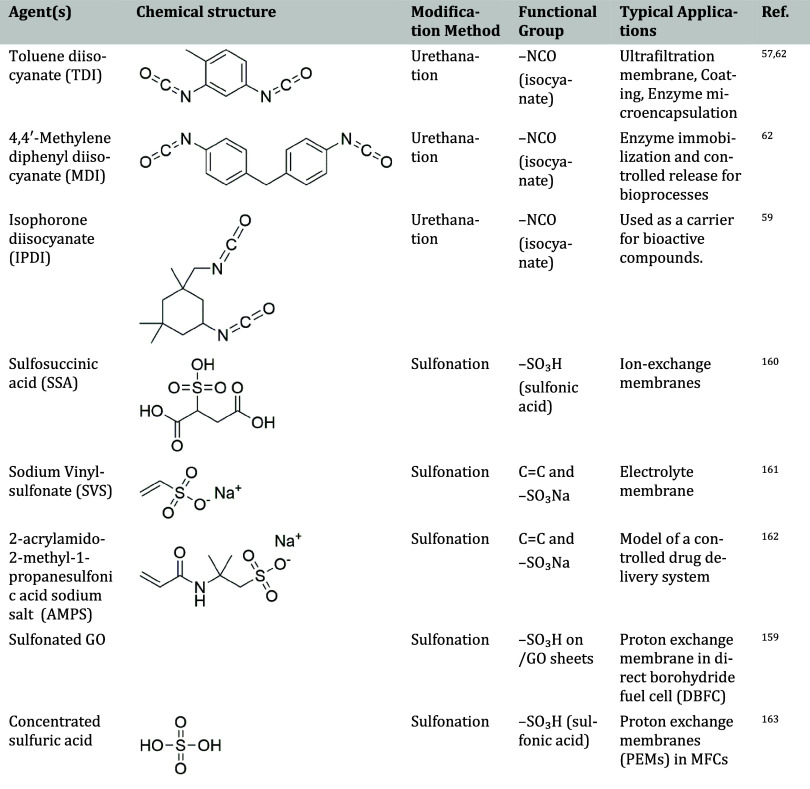
Applications and Functional Groups of PVA Modification Agents

In addition to urethanation and sulfonation, PVA can be phosphorylated to enhance hydrophilicity, ion conductivity, and metal ion complexation. This is typically achieved through condensation or ring-opening reactions with agents like phosphoric acid, trisodium trimetaphosphate (STMP), or sodium hexametaphosphate (SHMP) in aqueous media at 80–120 °C, sometimes with catalysts or pH adjustment.^164–166^ The resulting phosphate groups expand PVA’s utility in ion-exchange membranes, water purification, and biomedical applications.

Similarly, amination introduces amino groups onto the PVA backbone via nucleophilic substitution commonly under controlled temperatures. The resulting aminated PVA exhibits enhanced biocompatibility and reactivity, making it suitable for use in biosensors, enzyme immobilization, and bioactive membranes. To further highlight the versatility of PVA functionalization, various agents used in phosphorylation and amination reactions are summarized in Table 11.

**Table 11:**
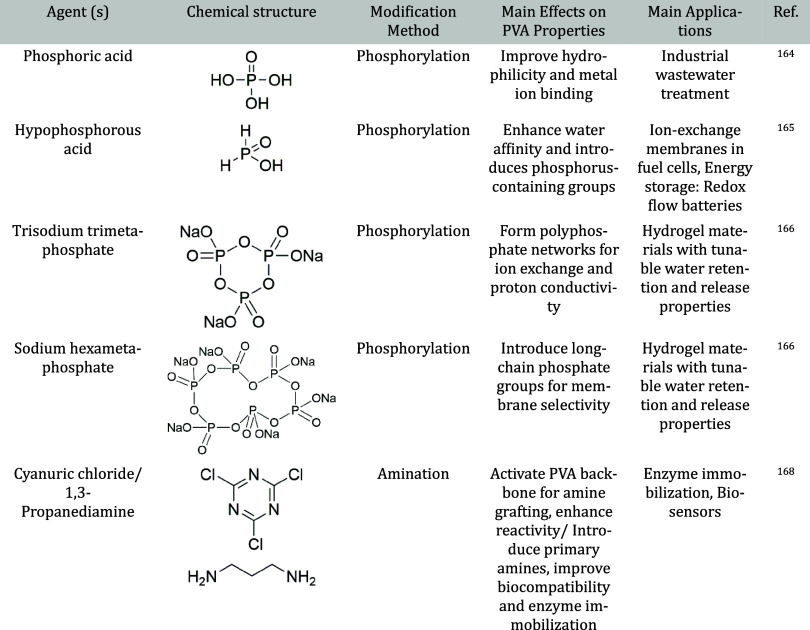
Main Effects and Applications of PVA Functionalization Agents

Urethanation improves thermal and mechanical properties but reduces water solubility and involves toxic isocyanates. Sulfonation boosts hydrophilicity and conductivity but may cause degradation under a high ionic strength. Phosphorylation enhances metal binding and solubility but requires harsh conditions and may lead to functional group leaching. Amination adds biocompatibility but has low efficiency and is temperature-sensitive.^167^ Thus, the method selection should align with application needs and processing limits.

The selection of a modification technique should consider the trade-offs between property enhancement and process complexity, tailored to the demands of each target application.

## Applications of Chemically Modified PVA

The applications of chemically modified PVA demonstrate its versatility across biomedical, engineering, and environmental fields due to significant functional enhancements. These advances underscore the critical role of chemical modification in expanding PVA’s potential and transforming it into a multifunctional material for cross-disciplinary innovations.

### Biomedical Applications

Cross-linking PVA with small chemical agents (citric acid,^169^ boric acid,^170^ borax^171^) or natural polymers (chitosan, gelatin, collagen, silk sericin^67,171,172^ ) via thermal esterification or genipin produces hydrogels with superior mechanical strength, elasticity, water retention, and biocompatibility. At the nanoscale and in core–shell architectures, cross-linking PVA with nanosilica (with or without chitosan),^172,173^ polyacrylamide–silk fibroin–chitosan composites,^174^ or gelatin-shell/PVA-core fibers^175^ imparts self-healing behavior, excellent biorecognition, and fiber stability. These systems are ideal not only for tissue-engineering scaffolds, wound coverings, and soft bioelectronic sensors but also for 3D-printed soft tissues and an intelligent drug-delivery platform.

Grafting PVA with natural polymeric chains enhances thermal stability, hydrophilicity, pH-responsive swelling, and reduces contact angle, while improving mechanical performance when swollen.^176–179^ These hydrogels find use in controlled drug delivery, wound dressings, and biodegradable tissue scaffolds, leveraging a high biocompatibility and tunable network architecture.^180–186^ Grafting small molecules onto PVA reduces fiber diameter, increases flexibility, crystallinity, and thermal stability, ideal for pH-responsive drug carriers and load-bearing wound-dressing materials.^53,95^

Grafting synthetic monomers and dendrons allows control of *T*_g_, amphiphilicity, degradation rate, and solubility. This has yielded thermoresponsive nanogels, antimicrobial patches, small interfering RNA (siRNA)-delivery nanoparticles, and tissue-engineering scaffolds with tunable degradation, well-suited for advanced biomedical uses such as 3D bioprinting and smart implantable devices.

Esterification of PVA with acids and anhydrides effectively lowers crystallinity, increases tensile strength, enhances thermal stability, and reduces swelling ratios.^49–51,54,137^ These materials have been employed in self-healing hydrogels,^51^ fullthickness wound dressings,^49^ the ability to control the release of nisin, helping to prevent infections or protect wounds, has the potential for use in the medical field,^54^ as well as controlled-release systems such as cinnamate-grafted PVA nanoparticles for poorly soluble drugs,^142^ lauroate-modified PVA hydrogels exhibit markedly enhanced friction and lubrication properties, making them highly suitable for artificial joint membranes.^144^ Meanwhile, acetalization using aldehydes decreases solubility and swelling, increases tensile strength, and boosts thermal stability up to 121 °C.^153,154^ These improvements facilitate controlled tissue engineering, drug release, high-temperature-resistant wound dressings, and protective coatings for medical devices.

Urethane formation reduces crystallinity, increases flexibility and tensile strength, and generates a multiporous microstructure ideal for enzyme immobilization and stable drug encapsulation.^60,62^ Sulfonation enhances the ion-exchange capacity and proton conductivity. The resulting materials are well-suited for biosensing applications and ion-uptake systems.^162,163^ Other methods, such as phosphorylation to form heavily cross-linked phosphate networks, yield pH-responsive, highly water-retentive gels that are ideally suited, and hold great promise for use in soft tissue scaffolds and other biomedical hydrogel applications.^166^ Amination with ethylenediamine replaces −OH with −NH_2_, improving enzyme (HRP, ADH) immobilization efficiency and catalytic stability in biosensor platforms.^167,168^

In summary, chemical modifications convert PVA from a basic water-soluble polymer into a highly versatile material. This adaptability supports a wide range of advanced applications, including smart drug delivery, self-healing wound dressings, biosensors, and 3D-printed tissue scaffolds, positioning modified PVA as a key material in next-generation biomedical and engineering technologies.

### Materials & Engineering applications

In the field of engineering materials, cross-linking of PVA enhances mechanical strength, thermal stability, and swelling control. For example, thermal esterification of PVA with citric acid, combined with clove oil, results in antimicrobial food packaging films with improved water resistance and moisture absorption, extending food shelf life.^69^ Additionally, ionic bonding between PVA and boric acid forms high-strength polymer fibers and elastic gels suitable for technical textiles and reinforced composites.^46,170^

In biobased materials and sustainable packaging, PVA–borate complexes are utilized as eco-friendly adhesives, supporting their use in wood processing and biocomposite applications.^45^ Moreover, the combination of silk sericin, a byproduct of the silk industry, with PVA and TGase produces biodegradable biofilms, offering a sustainable alternative to synthetic plastics and contributing to the circular economy.^187^ In soft electronics, PVA serves as a biocompatible ion-conductive matrix in flexible hydrogels with shape-memory and self-healing properties. Interpenetrating polymer networks (IPNs) between PVA and biopolymers create elastic, conductive, and antimicrobial gels ideal for wearable sensors and soft actuators.^172,173^ Beyond full IPNs, semi-interpenetrating polymer networks (semi-IPNs) offer another versatile platform for designing functional hydrogels. For instance, electrospun fibrous hydrogels based on PVA and a conductive polythiophene derivative (P3KBT) form conductive semi-IPNs, where P3KBT is physically entrapped within a cross-linked PVA network. These systems, which can be stabilized using green cross-linking methods such as thermal treatment or ethanol immersion combined with heating, exhibit excellent conductivity, photothermal responsiveness.^188^

Grafting PVA with tailored functional groups enables precise control over its mechanical, thermal, and barrier properties. For instance, covalent attachment of chitosan–procyanidin via CDI enhances elongation and reduces moisture permeability for biodegradable food packaging films,^94^ while grafting BTDA with polyphosphoric acid improves flexibility and reduces brittleness in preservation films.^189^ Beyond separation and packaging, free-radical grafting of poly­(oxyethylene methacrylate) (POEM) with CAN enhances CO_2_ permeability in gas-separation membranes,^110^ provides thermoresponsive adhesion with improved strength and heat resistance.^108^ Additionally, grafting acrylic acid improves thermal stability and swelling capacity in water-soluble binders for silicon anode batteries,^107^ boosts moisture resistance in paper coatings.^190^ These examples demonstrate grafting’s versatility in customizing PVA for a variety of advanced-material applications.

Esterification of PVA yields a suite of high-performance functional materials, potential applications in advanced technologies such as optical sensors and thermal insulation materials due to its optical stability and improved thermal resistance^137^ and self-healing hydrogels for soft electronics and sensors^51^ to ion-selective filtration membranes. Antimicrobial membranes with enhanced mechanical properties for food packaging and biomedical applications have been successfully developed,^54,136^ while membranes exhibiting low swelling, reduced free volume, and high pervaporation selectivity become ideally suited for liquid–mixture separation and purification applications, notably for ethanol–water separation,^138^ exemplified by nanofiber/net membranes from PVA–formic acid that remove 99% of 0.2 μm polystyrene particles at a flux of 3773 L/m^2^·h.^191^ Proton-exchange membranes for fuel cells exhibit outstanding conductivity to meet green-energy demands,^133^ and while the application potential of water-soluble photoresist films, enabled by aromatic ester groups such as benzoyl and cinnamoyl grafted onto the polymer backbone and of photoresponsive/sensing membranes opens up new opportunities in optoelectronics and printing technologies.^143,145^

Acetalization of PVA enhances its mechanical strength, thermal stability, and moisture resistance. For example, PVA modified with 1-naphthaldehyde (NA) and 9-ethyl-3-carbazolecarboxaldehyde (ECZA) shows that electron-donating groups (naphthyl, anthryl, carbazolyl) increase *T*_g_ and significantly enhance thermal stability, supporting the preparation of poly­(vinyl acetal)­s containing electron-donor moieties for electrically conductive polymeric compositions.^146^ Long-chain aldehydes such as 10-undecenal^149^ and 2-octyldodecanal^152^ improve flexibility and surface hydrophobicity.

Acetalized PVA also enhances optical and electronic properties, enabling applications in ion-conducting membranes and electronic materials. Aldehydes like 9-ethyl-3-carbazolecarboxaldehyde^146^ and benzaldehyde^151^ improve thermal stability and ion transport properties, suitable for alkaline water electrolysis (AWE) applications. Additionally, modifications using aldehydes create membranes with enhanced chemical and optical stability, expanding their use in electronic materials.^151^ Modifications using ketones yield poly­(vinyl ketal)­s, particularly poly­(vinyl acetone ketal) (PV-A-K), which can hydrolyze under humid conditions to regenerate PVA and a harmless ketone. This ability makes them especially suitable for water- or marine-degradable packaging, thereby reducing microplastic pollution. biodegradable packaging.^156^

The urethane modification of PVA improves tensile strength and increases thermal degradation temperatures.^58,62^ Additionally, it enhances fouling resistance,^57^ adjusts rheological behavior in lubricant formulations,^61^ and controls wettability and surface tension via urethanization with hydrophobic moieties (e.g., octyl isocyanate), enabling precise tuning of wetting properties, making it ideal for water-repellent or antifouling coatings.^192^

In contrast, the sulfonation of PVA via sulfuric acid esterification or Michael addition of vinylsulfonate monomers produces high-performance ion-exchange membranes. For example, sulfopropyl acrylate (SPA)-sulfonated, glutaraldehyde-cross-linked PVA achieves ∼16 mS/cm proton conductivity and ∼93% oxidative stability, making it a viable Nafion117 substitute in direct borohydride fuel cells (DBFCs).^161^ H_2_SO_4_-esterified PVA forms direct methanol fuel cell (DMFC) membranes with high proton conductivity and low methanol crossover.^158^ Beyond fuel cells, sodium vinylsulfonate-grafted PVA yields single-ion-conductor membranes with superior Li^+^ transport and solid electrolyte interphase (SEI) stability for lithium batteries.^162^

These modifications allow PVA to be tailored for enhanced mechanical strength, ion conductivity, water resistance, fouling resistance, and controlled release of the active compounds. Due to its structural design flexibility, modified PVA has become an ideal foundation for advanced materials in energy, environmental, biomedical, and industrial applications.

### Environmental and Other Applications

Functional modification of PVA with chelating groups such as dithiocarbamate has opened new avenues for its application in wastewater treatment, enabling efficient and selective removal of toxic metal ions such as arsenite from aqueous environments. The PVA material cross-linked with dithiocarbamate groups forms a dithiocarbamate-functionalized and cross-linked poly­(vinyl alcohol) (DFCPVA) aerogel with a porous structure, high surface area, and strong metal ion binding ability—key factors that enhance its arsenite (As­(III) removal performance.^193^ With features such as a high sorption capacity in both acidic and neutral conditions, a rapid adsorption rate, stability under cold storage, and a chelation-based sorption mechanism, this material is a promising candidate for arsenic removal from wastewater and groundwater. Moreover, the highly porous hybrid hydrogels synthesized via surface esterification of PVA acetal with pH or temperature-sensitive acrylic hydrogels show potential as efficient adsorbents for industrial wastewater treatment, offering high water retention, enhanced mechanical properties, superior adsorption rates and capacities, and low cost.^148^

Additionally, durable cross-linked networks (e.g., ethylene glycol diglycidyl ether (EGDE)^40^) allow PVA-chitosan hydrogels to maintain high performance over multiple reuse cycles. In solvent separation, cross-linked PVA forms membranes that selectively permeate water from azeotropic mixtures, aided by a porous structure and hydrophilic – SO_3_H groups, with high water selectivity (separation factor of 3452).^176^ Moreover, phosphorylated PVA^164^ enables oil/water separation with an efficiency exceeding 99.2%.

In the field of biobased packaging, modified PVA provides functionalities such as antibacterial activity and biodegradability, aligning with environmental protection goals. Grafting cinnamaldehyde onto PVA imparts good antibacterial activity at 2% cinnamaldehyde (CIN),^194^ making it suitable for food preservation. Meanwhile, modifications with syringaldehyde^150^ or glutaric acid^54^ produce thermally stable PVA films capable of biodegradation under acidic conditions or microbial activity, offering a sustainable alternative to conventional plastics.

In addition, chemically modified PVA also has other applications in the food and biotechnology industries as well as in cosmetics and functional materials. Chemically modified PVA with diisocyanates is widely used for enzyme encapsulation, enhancing stability and enabling controlled release.^62^ PUU (polyurethaneurea) microcapsules synthesized from PVA and dicyclohexylmethane diisocyanate (H12DI) exhibit high storage stability of immobilized *Bacillus subtilis* maltogenic amylase (BsMa) after 28 days of preservation, making them suitable for high-maltose syrup production or extended drug-delivery systems^60^ or extended drug-delivery systems. Notably, hexamethylene diisocyanate (HMDI)-derived microcapsules^62^ possess a flexible structure and excellent biocompatibility, ideal for enzyme immobilization in pharmaceutical and food industries.

Aromatic aldehydes modify PVA into high-gloss polymers for lipsticks and nail polishes.^152^ These materials reduce water solubility by replacing hydroxyl groups with aromatic acetal structures, while improving thermal stability. Remarkably, cinnamaldehyde-modified PVA^194^ forms antibacterial films for food packaging, inhibiting microbial growth and enhancing mechanical strength. Additionally, 4-hydroxybenzaldehyde-derived PVA achieves a glass transition temperature of 157 °C, making it suitable for heat-resistant coatings or biodegradable packaging.^150^

## Breakthroughs, Challenges, and Future Directions

### Breakthroughs

In recent years, significant breakthroughs have emerged, enabling enhanced performance and expanded applicability of PVA in areas such as biomedical engineering, packaging, and mechanical uses. These advancements stem from innovative chemical modifications, novel approaches, and nanotechnology integration, which have collectively transformed the potential of this longstanding polymer.

One of the most popular techniques for creating biomaterials nowadays is electrospinning. This process is regulated by variables like voltage, flow, and distance, which enable the modification of physical, chemical, and biological properties.^195,196^ Electrospinning can generate fine polymeric fibers with diameters reaching nanoscale.^197^ Moreover, the process is straightforward, simple, economical, and does not require high temperature—which is crucial for heat-sensitive materials.^198^ Because of their high porosity, tiny pore size, and large surface area, electrospun nanofibers are beneficial for a variety of uses.

Orozco et al. (2024) has utilized this technique to develop PVA/Gelatin/Heparin fibrillar scaffolds that are able to mimic morphological characteristics of native tissues, before using chemical cross-linking and heparin incorporation to further enhance the properties of the biomaterial.^199^ The electrospun fibers formed randomly in orientation without the presence of bead-like defects and interconnected pores,^200^ after cross-linking, they fused to form a nanoscale mesh. The chemically cross-linked scaffolds using glutaraldehyde showed increased hydrophobicity, as verified by greater wet contact angle and decreased degree of solubility. It was also determined that the PVA/G/H fibrillar scaffolds have characteristics that would make them suitable for usage in biological applications through mechanical testing and thermogravimetric analysis.^199^

Cross-linking of PVA is still being studied extensively by researchers, in search of new applications. A group of Mexican scientists has noticed the scarcity of optical characterizations of hydrogels, and from that, they have presented key breakthroughs in the development of optically functional, biocompatible hydrogels through the integration of genipin (GEN) cross-linking and multiwalled carbon nanotube (MWCNT) fillers into chitosan/poly­(vinyl alcohol) (CS/PVA) polymeric matrices. A 22–27% reduction in transmittance over the UV–Vis–NIR range and an increase in refractive index resulted from the incorporation of MWCNTs, which also improved crystallinity, added charge carriers, and scattering centers. On the other hand, GEN cross-linking improved the amorphous nature and created localized states that boosted optical conductivity (σ_opt_) and dielectric loss (*ε*″) while dramatically lowering the edge band (*E*_e_) by up to 33%. These results demonstrate that the optical and dielectric properties of materials may be precisely manipulated by varying their chemical composition, opening the door for specially made hydrogels for photonic, optoelectronic, and sensor applications.^201^

In another study, a detailed study of PVA/Boric acid (BA) hydrogels carried out by the Department of Astronautical Electrical and Energy Engineering at Sapienza University of Rome revealed that this PVA-based material is well-suited for integration into astronaut personal protective equipment.^202^ BA was chosen as a cross-linking agent to enhance both the mechanical strength and radiation shielding capabilities of the gels. This resulted in the development of cross-linked PVA/BA gels with superior features in terms of overall volume, weight, and radiation shielding efficacy when compared to the liquid water garments currently employed in space missions.^202^ The synthesized material possessed nearly double the storage modulus compared to PVA, and a shielding effect comparable with liquid water.

A novel dual cross-linking strategy was carried out by combining in situ and ex situ cross-linking methods as well as activating with high-concentration KOH, which significantly enhances the mechanical properties and thermal stability of quaternized poly­(vinyl alcohol) (QPVA) membranes while maintaining high ionic conductivity.^203^ Using glutaraldehyde as cross-linker, the QPVA membranes’ properties were evaluated at different KOH concentrations, ranging from 5 to 8 M. The M1 membrane, prepared with 5 M KOH activation, achieved the highest ionic conductivity (40.93 mS/cm before equilibrium, 33.41 mS/cm after equilibration) and moderate oxidative stability (81.10%). It was concluded that this approach effectively suppressed swelling and enhanced mechanical stability, although it somewhat reduced the oxidative stability. These optimizations provide a path for developing durable, high-performance anion exchange membranes for fuel cells and electrolyzers.

Wu et al. (2025) investigated the impact of temperature and microwave irradiation on the structure of PVA in an aqueous solution.^204^ Using Raman spectroscopy and atomic force microscopy (AFM), they observed that increasing temperature disrupted the tetrahedral hydrogen bonding between PVA and water, shifting it to a chain-like structure, with temporary enhancement at 313.15K. Microwave irradiation increased intra- and intermolecular hydrogen bonds, but after 180 s, hydroxyl groups were removed, causing bond loss. This research highlights the potential of using microwave irradiation and temperature control to fine-tune the properties of chemically modified PVA.

In conclusion, recent advancements in research to improve the chemical properties of PVA often focus on new techniques to overcome the initial limitations of classical methods. With the development of technologies, such as the increasing popularity of electrospinning technology, the modification of PVA holds great potential, as its applications become more diverse and extensive. In the current context, cross-linking, characterized by its ease of execution, stands out as the most attractive method, with many groundbreaking advancements being made. Needless to say, grafting and functionalization of PVA are also benefiting from technological progress^53^ and the expansion of multidisciplinary applications.^205^

### Challenges

Chemical modification strategies for PVA, while offering significant potential to enhance the properties of the polymer, also face several notable challenges. These challenges include toxicity concerns, control over reaction parameters, impact on biocompatibility, processing complexity, and environmental impacts.

GA is one of the most commonly used cross-linking agents; however, it may leave toxic residues in the material. This poses significant challenges, especially in the biomedical field, where biocompatibility is a critical requirement.^36,187^ Moreover, the degree of cross-linking is not yet tightly controlled; excessive cross-linking can lead to brittleness and substantially reduce the material’s flexibility in applications.^71^

The grafting method sometimes faces challenges in achieving high efficiency due to insufficient control over influencing factors such as reactant concentration, temperature, and solvent composition, all of which significantly affect both the reaction yield and the properties of the modified polymer. Moreover, although this method can enhance mechanical and/or thermal stability, it may reduce the material’s versatility and biocompatibility.^206^

Functionalizing PVA to achieve the desired properties requires strict control of the reaction parameters. However, chemicals such as isocyanates and acid chlorides, used in the functionalization process, can pose significant toxicity risks and have a negative impact on the environment.^176^ Moreover, the functionalization process can reduce the inherent hydrophilicity and swelling capacity of PVA, especially when hydrophobic groups are attached, which is not suitable for applications that require high hydrophilicity and good swelling properties, such as in biomedical systems or materials that need to retain moisture.^65^

In conclusion, while modifications of PVA through grafting, cross-linking, and functionalizing prove their potential in enhancing material properties, challenges pertaining to toxicity, complexity in processing, and proficiency still stand. Future studies should shift the focus to optimizing the techniques involved in order to mitigate pollution, improve scale-up probabilities, and ensure long-term biocompatibility for various industrial as well as medical applications.

### Future Directions

The development of PVA chemical modification is driven by new scientific and technological demands. Traditional methods, though effective, face limitations in sustainability, multifunctionality, and precision. Future research will focus on greener processes, better property control, and the expansion of PVA into more complex materials.

One of the most pressing demands in the future development of PVA chemical modification is the transition toward environmentally friendly and sustainable processes. As such, there is an urgent need to replace conventional reagents with greener alternatives, enzymatic catalysis, and aqueous-phase reactions. Additionally, efforts are being directed at developing solvent-free, microwave-assisted, or low-energy cross-linking techniques to minimize production cost, energy consumption, and processing time.

Future research on electrospinning using chemically modified PVA should focus on the concurrent design of the fiber morphology and chemical functionality. The integration of advanced electrospinning techniques, such as coaxial or triaxial spinning, with physical or chemical cross-linking methods can significantly enhance the performance of scaffolds in applications. The integration of chemically modified PVA into high-throughput electrospinning platforms, such as needleless systems or continuous production setups, will be critical for successful industrial-scale technology transfer.

An important future research direction is the comprehensive evaluation of the performance of PVA-based systems under real-world and harsh environmental conditions, such as applications in marine environments or outer space. Assessing the durability, stability, and functional retention of these materials in each environment, across extreme temperature ranges or under the influence of strong corrosive agents will be crucial for expanding their practical applications.

Moreover, the integration of advanced computational tools such as diffusion models, FDTD simulations, and data-driven optimization algorithms is opening up new approaches for the design and optimization of PVA materials.

## Conclusions

Poly­(vinyl alcohol) (PVA) is recognized as a versatile material due to its distinctive film-forming capabilities and biodegradability, facilitating extensive applications across biomedicine and environmental technology. Nonetheless, its intrinsic drawbacks, such as low thermal stability and limited mechanical strength, require ongoing chemical modifications to satisfy the increasing demands of advanced applications.

This review outlines various strategies employed to augment the functional properties of PVA, including cross-linking, grafting, and functionalization. Cross-linking is the most straightforward method, often resulting in enhanced mechanical strength, stability, and resistance to degradation. Grafting techniques permit a higher degree of customization of additional properties, such as compatibility and adjustable hydrophilicity, by incorporating diverse functional groups, each possessing unique characteristics. Functionalization further permits accurate adjustment of bulk and surface properties, increasing PVA’s utility in areas like sensing, drug delivery, and a variety of environmental applications.

PVA is currently utilized across a wide range of industrial and scientific fields due to its ease of handling and biocompatibility. In the biomedical sector, PVA is employed in self-healing wound dressings, smart drug-delivery systems, biosensors, and tissue-engineering scaffolds, where its nontoxicity and versatility after chemical modifications are particularly advantageous. In packaging, PVA-based films are valued for their biodegradability, moderate heat resistance, and gas barrier properties, making them suitable for eco-friendly food packaging and single-use plastics. PVA, with its unique ability to form hydrogels, also plays a key role in water treatment, adhesives, paper coatings, and textiles. Moreover, its compatibility with nanomaterials has enabled the development of advanced composites for use in electronics, ion-exchange membranes, and fuel cells. These diverse applications highlight PVA’s adaptability and underscore its growing relevance in both traditional and emerging technologies.

The incorporation of nanotechnology and innovative techniques, such as electrospun nanofibers and dual cross-linking membranes, has further elevated PVA’s performance. These approaches have not only enhanced its strength and durability but also enabled multifunctionality. Concurrently, greener modification processes, including solvent-free systems and microwave-assisted grafting, are making PVA a more sustainable option for large-scale applications.

Despite these advancements, drawbacks remain in the complexity of synthesis, longevity, and environmental safety, particularly when chemical reagents are used in modification. Future research should focus on integrating sustainable practices with high-precision design, supported by developed computational tools and real-world performance evaluations in extreme environments.

With continued innovation and interdisciplinary efforts, modified PVA has the potential to become a high-performance material—versatile, eco-friendly, and well-suited material for critical applications in medicine, packaging, electronics, and environmental technologies.

## Abbreviations

CCR2: CC chemokine receptor 2
CCL2: CC chemokine ligand 2
CCR5: CC chemokine receptor 5
TLC: thin layer chromatography
